# PSGRN: Gene regulatory network inference from single-cell perturbational data through self-training with synthetic gold standards

**DOI:** 10.1126/sciadv.aeb3376

**Published:** 2026-04-29

**Authors:** Xinhan Song, Kaiwen Deng, Mandy Chen, Yuanfang Guan

**Affiliations:** ^1^ Department of Computational Medicine and Bioinformatics, Michigan Medicine, University of Michigan, Ann Arbor, MI, USA.; ^2^ College of Literature, Science, and The Arts, University of Michigan, Ann Arbor, MI, USA.; ^3^ Department of Internal Medicine, University of Michigan, Ann Arbor, MI, USA.

## Abstract

Gene regulatory networks (GRNs) are essential for understanding how genes coordinate cellular processes. Large-scale single-cell perturbation studies now offer powerful opportunities for GRN inference, yet many state-of-the-art (SOTA) methods fail to fully use interventional information. We present PSGRN, a top-performing method in the CausalBench Challenge, which integrates interventional and observational single-cell RNA sequencing data using a self-training framework with synthetic gold standards. Across eight datasets and six evaluation metrics, PSGRN consistently outperformed existing approaches. With interventional data, it achieved up to 43% higher Wasserstein distances and the lowest false omission rate in K562 compared with recent SOTA methods. Using experimentally validated regulatory interactions, PSGRN showed up to 30% gains in precision and over 100% gains in recall. These results highlight PSGRN’s versatility and scalability, establishing it as a robust tool for GRN inference and biological discovery from single-cell data.

## Teaser

A self-training strategy boosts discovery of gene regulatory networks from single-cell perturbations.

## INTRODUCTION

Understanding the causal relationships between gene expression and constructing gene regulatory networks (GRNs) has a profound impact on therapeutic design and drug efficacy (*1*, *2*). Recent advances in single-cell high-throughput sequencing have transformed this field, enabling the simultaneous analysis of thousands of genetic perturbations in a single experiment and expanding the scope of causal inference in cellular systems (*3*–*5*).

Inferring the network structure from such data has been widely studied in both computer science and bioinformatics. Recent GRN inference methods viewed this question as a feature-ranking problem. For instance, GENIE3 and its successor GRNBoost predict the expression of individual genes based on the expression of all other genes, ranking regulatory relationships according to feature importance scores (*6*–*8*). Other approaches, particularly in the causal discovery domain, introduced continuously differentiable constraints, making them suitable for deep learning and gradient-based optimization. For example, DCDI encodes the directed acyclic graph (DAG) structure as a binary adjacency matrix and applies continuous constraints, optimized using stochastic gradient descent (*9*). These methods enable inference on larger and more complex datasets that combine observational and interventional data.

Despite these advancements, substantial performance gaps have emerged when evaluating these methods on a newly introduced single-cell perturbation benchmark dataset, CausalBench (*10*). GRN inference methods, originally designed for observational data, often fail to improve when interventional data are incorporated. Moreover, cutting-edge causal discovery algorithms show limited gains despite the availability of additional perturbation data. This contrasts with the common assumption that interventional data should enhance causal inference. These limitations may arise from training the models on indirect gold standards (e.g., the gene expressions), and evaluating them with unstable metrics (e.g., feature importance scores), or on synthetic testing datasets. Thus, retrieving a reliable annotation that reflects direct gene-gene interactions and training models under its supervision may be crucial for advancing GRN inference in single-cell perturbation studies.

In this work, we introduce PSGRN, a semisupervised tool that integrates the correlation information from both observational and interventional data to infer GRNs with a self-training framework. Correlations between gene expressions can efficiently capture regulatory relationships (*11*), and synthetic, pseudo–ground truths derived from these correlations were used iteratively to refine the model through self-training (*12*). This self-training framework enables PSGRN to iteratively denoise and improve the initial pseudolabels, allowing it to learn higher-order regulatory patterns beyond simple correlation. This iterative process allows the model to progressively correct noisy pseudolabels and extract more reliable regulatory signals. By combining these advantages, PSGRN achieves enhanced performance and robustness in capturing true gene regulatory relationships. This approach led to PSGRN winning the 2023 GSK.ai CausalBench Challenge (*13*), demonstrating its effectiveness in this competitive evaluation.

## RESULTS

### Data collection and self-training with pseudoannotation to infer GRNs

Two single-cell datasets of the K562 cell line and the RPE1 cell line were provided as part of the challenge for algorithm development. They were retrieved from a genome-scale Perturb-seq experiment with CRISPR perturbations (*14*). Each dataset organized its expression data in a matrix, where the columns represent the measured genes and the rows represent the cells. Each cell had a label indicating which gene was perturbed in that cell (the interventional data) or whether there was no targeted gene (marked as nontargeting; the observational data).

**Fig. 1.**
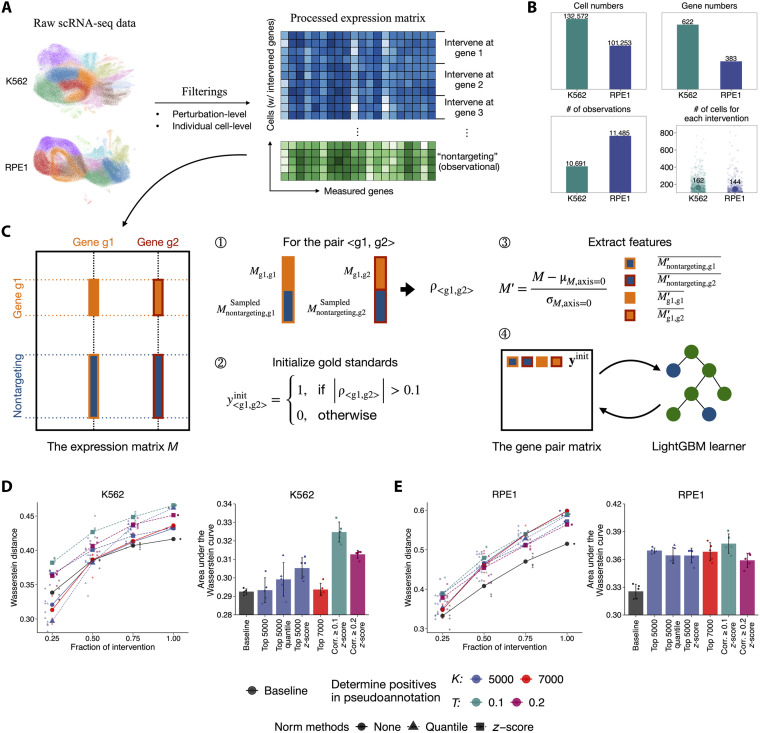
Experimental design, data processing, and performance evaluation of PSGRN. (**A**) Preprocessing of K562 and RPE1 Perturb-seq datasets. Perturbations were filtered on the basis of differential expression, cell counts, and knockdown efficiency, and cells were filtered by comparing target gene expression to nontargeting controls. The processed data were organized into cell-by-gene expression matrices with labels indicating the perturbed gene or observational status. (**B**) Summary statistics of the filtered datasets, including the total numbers of cells and genes (top), the number of observational cells (bottom left), and the distribution of cells per perturbation (bottom right). The median number of cells per perturbation is indicated; eight perturbations with more than 1000 cells are omitted for clarity. (**C**) Overview of the PSGRN framework. Gene-gene correlations are used to generate pseudoannotations and extract pairwise features, which are iteratively refined through a self-training process to infer regulatory interactions. (**D** and **E**) Parameter selection for pseudoannotation generation and feature normalization. Wasserstein distances are shown across different fractions of interventional data, along with the corresponding areas under the curves. “Baseline” denotes selecting the top 1000 correlated pairs without training. “5000” and “7000” indicate the number of top-correlated pairs used as initial positives, and “0.1” and “0.2” denote correlation thresholds. Points represent results from random subsampling of interventional data, with averages highlighted.

The raw expression matrix of K562 includes 310,385 cells with 2058 unique perturbations and 8563 genes, and that of RPE1 includes 247,914 cells with 2394 perturbations and 8749 genes. We are interested in the most significant regulatory relationships and focused on high-quality and effective perturbations. Therefore, a two-step quality control (QC) process was applied to the raw interventional data to select the strong and effective perturbations following the criteria described in the Perturb-seq experiment (*14*). At the perturbation level, we kept the perturbations that resulted in at least 50 differentially expressed genes (DEGs), had at least 25 high-quality living cells, and achieved at least 30% knockdown of their target gene. At the individual cell level, we excluded cells where the perturbed gene’s expression remained above the 10th percentile of its unperturbed control in the observational data. We also excluded perturbations with fewer than 100 effective cells. Throughout the QC process, unperturbed control cells (nontargeting) were retained as observational data (Fig. 1A). The combined data were then normalized by the total Unique Molecular Identifier (UMI) counts per cell across all detected genes and log transformed. Last, we subset the expression matrix to only keep the features (genes) corresponding to the effective perturbations (tables S3 and S4).

The processed K562 dataset maintains 622 unique perturbations across 132,572 cells and 622 genes, with a median of 162 cells per perturbation, whereas the RPE1 dataset consists of 383 unique perturbations across 101,253 cells and 383 genes, with a median of 144 cells per perturbation (Fig. 1B). The difference in gene numbers between the two cell lines may be due to variations in perturbation efficiency, with K562 perturbations being more effective, leading to the retention of more cells and associated perturbed genes. For model training and evaluation, we followed the settings of CausalBench, splitting the data at the level of individual cells stratified by intervention targets (including nontargeting). We used 80% of the data as the training set to infer the GRNs and evaluate them on the 20% held-out test data.

PSGRN was developed under the self-training framework. Given an expression matrix with interventional data, our tool infers the GRNs through the following steps. First, it generated the gene pairs from all measured genes (genes from the matrix columns) and assigned pseudoannotations based on the correlations calculated from their expressions. Next, for each pair of genes, we extracted the average expression values of the genes in the pair before and after the interventions. Then, these features are used to create a dataset along with the pseudoannotations. Last, a classification model was trained on the entire dataset, including all possible gene pairs and their pseudoannotations, and then used to predict the probabilities for these gene pairs on the same data (Fig. 1C). The top 1000 (PSGRN 1K) or 5000 (PSGRN 5K) gene pairs with the highest probabilities were inferred to have regulatory relationships.

The hyperparameters of our method included how to assign pseudoannotations for the gene pairs (i.e., annotating the first *K* top-correlated gene pairs or pairs with correlation larger than a threshold *T* as the positives), the number of negative samples selected for model training, as well as the additional normalization step before feature extraction. To choose the optimal set of hyperparameters, we conducted experiments on the training datasets with different fractions (0.25, 0.5, 0.75, and 1.0) of interventional data. Models were trained to infer 1000 regulatory gene pairs and compared with a baseline method that generated inferences by directly selecting the 1000 top-correlated gene pairs. We assessed the performance on test data at each fraction using the Wasserstein distance, which measures the expression difference in the target gene before and after perturbing its regulator. The area under the Wasserstein distance curve (AUC-Wasserstein) was used to summarize performance across all fractions (Fig. 1, D and E). A random gene pair is expected to have a score of zero in Wasserstein distances and AUC-Wasserstein.

The optimal hyperparameter settings (correlation threshold *T* = 0.1 and *z*-score normalization) were determined through preliminary experiments (table S1) and validated by repeating experiments five times with randomly sampled subsets of the interventional data (table S2). Under these conditions, PSGRN achieved an average AUC-Wasserstein of 0.3248 (± 0.005) on the K562 dataset and 0.3771 (± 0.01) on the RPE1 dataset, outperforming the baseline correlation–based method, which had an average AUC-Wasserstein of 0.2926 (± 0.001) and 0.3255 (± 0.008), as well as the DCDI methods (DCDI-G K562: 0.1045 ± 0.001; DCDI-G RPE1: 0.1032 ± 0.017; DCDI-DSF K562: 0.1047 ± 0.0003; DCDI-DSF RPE1: 0.0954 ± 0.001) and GRNBoost (K562: 0.1218 ± 0.002; RPE1: 0.1055 ± 0.003; fig. S1). These results also exceeded other tested hyperparameter groups by at least 3.83%. Thus, this optimized model was used for all subsequent analyses.

### PSGRN is robust to the fraction of intervention data and overall sample sizes

To benchmark PSGRN’s scalability and robustness with varying amounts of interventional data with the other methods, we trained the models and generated inferences using the training datasets with different fractions of interventional data, including two additional lower fractions (5, 15, 25, 50, 75, and 100%). In addition, to benchmark the scalability regarding sample size, we created six more training datasets by randomly subsampling data from both the observational and interventional datasets with these fractions. The inferences were evaluated on the test data. In addition to the Wasserstein distance, two other metrics were used for evaluation: statistical precision and the false omission rate (FOR). Statistical precision measures the ratio of gene pairs with significant Wasserstein distances to the total number of gene pairs, whereas FOR quantifies the absence of gene interactions by measuring the number of missed actual positives. An effective algorithm should balance maximizing the Wasserstein distance and minimizing FOR.

**Fig. 2.**
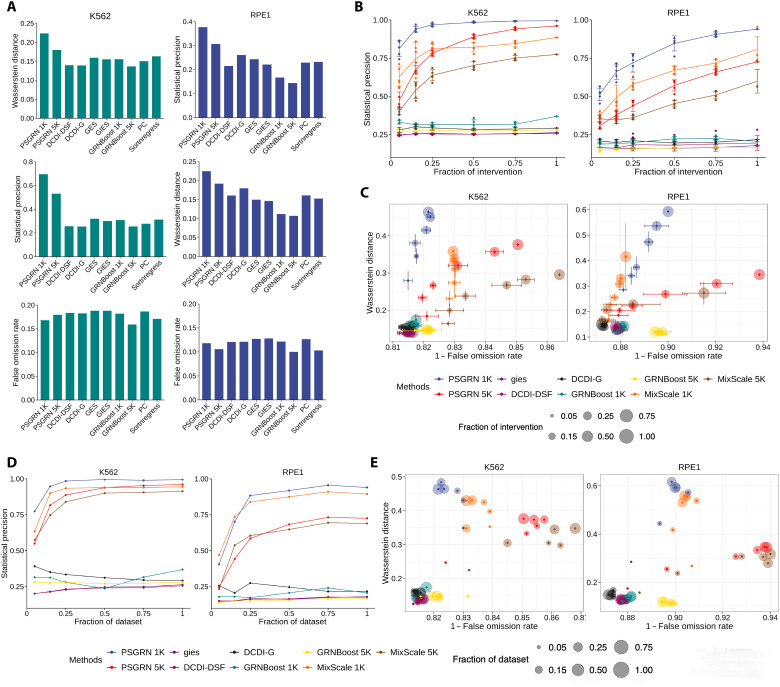
Benchmarking PSGRN against other GRN inference methods across different fractions of the interventional data and the entire datasets. (**A**) Performance of PSGRN (1K and 5K) and seven other algorithms on the K562 and RPE1 observational data. (**B**) Performance of increasing fractions of interventional data on the statistical precision of PSGRN (1K and 5K) and six other algorithms as the fraction of interventional data increases for the K562 (left) and RPE1 (right) datasets. Each point represents the mean precision across six random seeds. (**C**) Mean Wasserstein distance and 1 − FOR of PSGRN (top 1K and top 5K) and six other algorithms across increasing fractions of interventional data for the K562 (left) and RPE1 (right) datasets. (**D**) Statistical precisions of PSGRN (1K and 5K) and six other algorithms as the dataset size increases for the K562 (left) and RPE1 (right) datasets. (**E**) Performance of increasing fractions of dataset size on the mean Wasserstein distance and 1 − FOR of PSGRN (top 1K and top 5K) and the other six algorithms for the K562 (left) and RPE1 (right) datasets.

PSGRN demonstrated superior scalability in leveraging additional interventional data and benefited more substantially from increased intervention ratios than benchmark methods. Both PSGRN 1K and PSGRN 5K showed notable improvements in statistical precision as the fraction of intervention increased. In the K562 cell line, the precision improved from 0.8122 to 0.9950 for PSGRN 1K and from 0.4712 to 0.9612 for PSGRN 5K. In comparison, MixScale also benefited from additional interventional data but exhibited a slower rate of improvement, increasing from 0.6280 to 0.8850 (MixScale 1K) and from 0.4032 to 0.7744 (MixScale 5K), respectively. A similar trend was observed in the RPE1 dataset. Under full intervention, PSGRN outperformed MixScale by 12.4 and 24.1% in K562 (1K and 5K, respectively) and by 16.5 and 21.1% in RPE1 (Fig. 2B).

The superiority was also reflected in its favorable trade-off between higher Wasserstein distances and lower FOR as the fraction of intervention increased. The Wasserstein distance increased from 0.2794 to 0.4639 for PSGRN 1K and rose from 0.1842 to 0.3764 for PSGRN 5K. Among the benchmark methods, although MixScale showed substantial improvement with increasing intervention ratio, its performance was overall inferior to that of PSGRN. The remaining benchmark methods exhibited consistently weak performance with minimal improvements. Notably, under full interventional training in K562, PSGRN achieved markedly highest Wasserstein distances, exceeding MixScale by 29.5% (1K) and 27.4% (5K). A similar pattern was observed in RPE1, where PSGRN also substantially outperformed MixScale in Wasserstein distance, with improvements of 42.7% (1K) and 26.6% (5K), respectively. With respect to FOR, PSGRN achieved the lowest FOR among all methods in the K562 dataset (0.8215 for 1K and 0.8505 for 5K), outperforming both MixScale and GRNBoost. In the RPE1 dataset, MixScale achieved slightly lower FOR values (0.8822 for 1K) compared to PSGRN (0.9000 for 1K) (Fig. 2C). Similar trends were observed when scaling by overall sample size (Fig. 2, D and E). As the sample size increased, PSGRN consistently achieved larger Wasserstein distances and reduced FORs, especially in the RPE1 dataset.

Apart from the widely used machine learning–based inference methods, we also compared PSGRN with two baseline approaches: DEG analysis (*15*) and MeanDifference (*16*). DEG analysis represents a straightforward strategy that leverages the observed perturbation arms to infer regulatory relationships based on expression changes. MeanDifference, a top-performing method in the CausalBench Challenge, provides a simple yet competitive benchmark for causal inference. Compared to DEG, PSGRN achieved substantially higher precision across both datasets, with improvements ranging from 25 to 35%, highlighting its enhanced ability to extract meaningful regulatory signals (fig. S2). Although MeanDifference slightly outperformed PSGRN in K562, PSGRN delivered more robust performance in RPE1 and demonstrated a better balance between sensitivity and specificity, as reflected by its lower FORs.

To further benchmark the model’s ability to generalize to the unseen interventional cases, we designed another series of evaluations where the training and testing datasets were split at the level of perturbations. Models trained on part of the interventional data were tested on the remaining perturbations. Top inferences were selected from the pairs including the unseen perturbed genes. Differential expression analysis and the MeanDifference were not available under this setting as the test expression data were concealed from them. As a result, PSGRN showed similar superiority to GRNBoost, with an average 126.87% improvement in Wasserstein distance and 10.23% improvement in FOR in the K562 and RPE1 datasets (fig. S3).

PSGRN also maintained strong performance even without interventional data (Fig. 2A). In this scenario, PSGRN 5K achieved a Wasserstein distance of 0.1799 and a precision of 0.5314, whereas PSGRN 1K scored 0.2237 in Wasserstein distance and 0.695 in precision, outperforming the other algorithms by at least 66.17 and 117.32%, respectively. In the RPE1 dataset, PSGRN achieved a Wasserstein distance of 0.1917 (5K) and 0.2246 (1K) and precision values of 0.3066 (5K) and 0.3770 (1K), outperforming the best benchmark algorithm by over 17.52 and 44.50%, respectively. Although PSGRN’s FOR was slightly higher than GRNBoost, the overall results highlight PSGRN’s flexibility and strong performance across a wide range of data scenarios. Notably, DEG and MeanDifference are not applicable in the absence of interventional data, further emphasizing PSGRN’s adaptability in real-world applications.

### PSGRN recovers more experimentally verified interactions

In addition to the statistical evaluations, we conducted biological evaluations to assess the practical utility of PSGRN. To ensure the model’s predictions were biologically relevant and functionally valid, we retrieved the ground-truth networks from four datasets: CORUM, STRING Network, STRING Physical, and the cell type–specific chromatin immunoprecipitation sequencing (ChIP-seq) datasets (see Materials and Methods). These datasets provided a comprehensive pool of known gene interactions, which we integrated into a unified biological database containing 784,817 gene pairs for K562 and 784,405 pairs for RPE1 (fig. S4). These refined networks served as gold standards for biological evaluation. In addition to precision and recall, we computed F1 scores to assess how different top-K selections (1K versus 5K) influenced overall performance for PSGRN, GRNBoost, and MixScale. Moreover, to examine how self-training refines the initial correlation-based estimates, we compared the rankings of all ground-truth regulatory pairs before and after self-training (fig. S9). When full interventional data were available, PSGRN substantially elevated the ranks of biologically validated interactions, with mean rank shifts of −9446.56 in K562 and −4066.42 in RPE1. These shifts indicate that self-training systematically improves the prioritization of true regulatory pairs beyond what can be achieved by correlation alone.

**Fig. 3.**
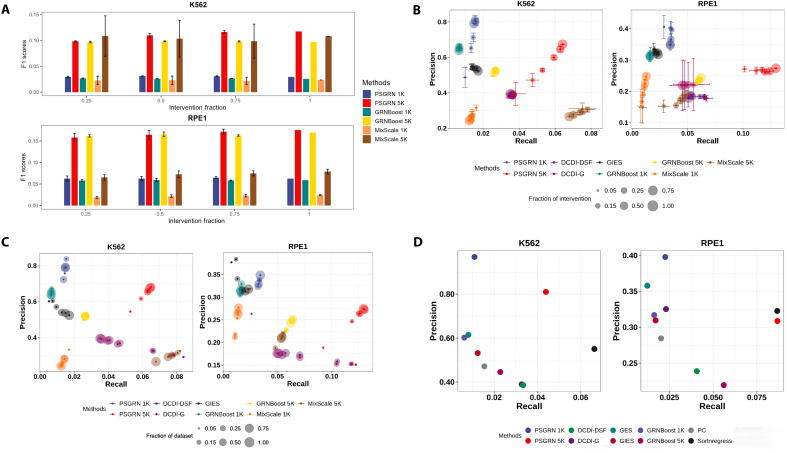
Benchmark PSGRN with the other GRN inference methods on the biological evaluation metrics trained with different fractions of intervention and dataset. (**A**) Biological F1 scores of PSGRN (top 1K and top 5K) and the other algorithms with increasing fractions of interventional data for the K562 (top) and RPE1 (bottom) datasets. Each bar corresponds to the mean F1 score of five independent experiments for each intervention fraction, with error bars representing the SD. (**B**) Performance of increasing fractions of interventional data on the biological precision and recall of PSGRN (top 1K and top 5K) and the other six algorithms for the K562 (left) and RPE1 (right) datasets. Each point corresponds to the mean precision of five independent experiments for each intervention fraction. (**C**) Performance of increasing fractions of dataset size on the biological precision and recall of PSGRN (top 1K and top 5K) and the other six algorithms for the K562 (left) and RPE1 (right) datasets. Each point corresponds to the mean precision of five independent experiments for each intervention fraction. (**D**) Biological evaluation performance of PSGRN (1K and 5K) and other algorithms on the K562 and RPE1 observational data in precision and recall.

The advancements of PSGRN’s scalability and efficiency to additional interventional data were maintained when evaluated against biological gold standards (Fig. 3B and fig. S6). As the level of interventional data increased, both PSGRN 1K and PSGRN 5K showed progressively better performance in terms of precision and recall. Selecting the top 5000 pairs consistently yielded higher F1 scores than selecting the top 1000 pairs across both datasets. With full interventional data, the F1 score of PSGRN 5K reached 0.1174 compared to 0.0293 for PSGRN 1K in K562 and 0.1753 compared to 0.0624 in RPE1. GRNBoost and MixScale showed the same pattern (Fig. 3A). In broader benchmarking on the K562 dataset, PSGRN 5K outperformed GRNBoost 5K by 30.2% in precision (0.6740 versus 0.5178) and 142.4% in recall (0.0642 versus 0.0265). Compared with MixScale, PSGRN 5K demonstrated an even more pronounced advantage in precision, exceeding MixScale 5K by 154.0% (0.6740 versus 0.2654) while showing a modestly lower recall (0.0642 versus 0.0678). On the RPE1 dataset, PSGRN 5K also achieved notable improvements over all other algorithms, outperforming the best baseline method by 12.7% in precision (0.2744 versus 0.2434) and 107.8% in recall (0.1293 versus 0.0622).

Unlike their relatively strong performance in statistical evaluations, MeanDifference failed to generalize to biological evaluations. It consistently underperformed compared to PSGRN across interventional data levels, achieving lower precision and recall across both K562 and RPE1 datasets. Similarly, DEG also showed inferior biological performance. Specifically, PSGRN achieved 20 to 45% higher precision and up to 170 to 270% higher recall compared to MeanDifference and DEG, highlighting its advantage in recovering biologically meaningful regulatory interactions (fig. S8).

As expected, PSGRN is also scalable to increasing sample sizes, consistently showing better results in the precision-recall trade-off than the other algorithms (Fig. 3C and fig. S7). With larger sample sizes, PSGRN 5K improved precision and recall by 63.6% (from 0.4120 to 0.6740) and 331.7% (from 0.0149 to 0.0642) in K562 and by 21.0% in precision (from 0.2268 to 0.2744) and 122.9% in recall (from 0.0580 to 0.1293) in RPE1, whereas other methods showed limited or no improvement.

When tested on observational data, PSGRN remained competitive (Fig. 3D and fig. S5). In the K562 dataset, although PSGRN 5K had 48.3% lower recall compared to GRNBoost 5K (0.0439 versus 0.0228), it outperformed GRNBoost 5K by 81.8% in precision (0.8101 versus 0.4456). In the RPE1 dataset, PSGRN 5K demonstrated 40.6% better precision (0.3086 versus 0.2194) while maintaining a similar recall to GRNBoost 5K (0.0868 versus 0.0561). These results suggest that, although PSGRN is optimized for interventional data, it still delivers robust performance in observational data, particularly in terms of precision.

### PSGRN recovers biologically meaningful regulatory networks consistent with DEG

**Fig. 4.**
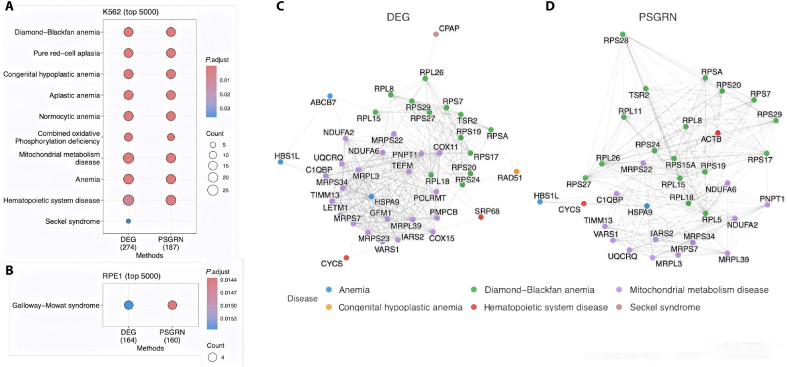
Comparison of disease term enrichment and gene interaction networks inferred by DEG and PSGRN on K562 and RPE1 datasets. (**A**) Enriched disease terms identified using the top 5000 gene pairs predicted by DEG and PSGRN on the K562 dataset. Dot color represents the adjusted *P* value (red: more significant), and dot size indicates the number of genes associated with each disease term. (**B**) Enrichment analysis on the RPE1 dataset. (**C**) Disease-associated gene interaction subnetwork from DEG-inferred results on K562. Nodes are genes, colored by disease category, and edges represent predicted regulatory links. (**D**) Subnetwork from the disease-associated gene interaction subnetwork from PSGRN-inferred results on K562.

To better assess the biological relevance of the predicted networks, we performed disease term enrichment and network analyses on the top 5000 interactions inferred by PSGRN and DEG using the K562 and RPE1 datasets (Fig. 4). In the K562 dataset, PSGRN identified a comparable or larger number of significant disease terms (10 terms with adjusted *P* value < 0.01) compared to DEG. Notably, many of the enriched terms identified by PSGRN overlapped with those captured by DEG, indicating that PSGRN can recover disease relevant signals consistently. The top enriched terms are associated with hematopoietic processes, including Diamond-Blackfan anemia, pure red cell aplasia, and hematopoietic system disease, which can reflect the leukemia-derived origin of K562. However, it should be noted that these signals may also reflect dominant, broadly coregulated modules, such as ribosomal and mitochondrial processes, which are commonly detected in large-scale expression and interaction datasets (Fig. 4A). In the RPE1 dataset, both methods significantly enriched Galloway-Mowat syndrome, although the overall smaller number of enriched terms likely reflects the nonhematopoietic nature of the cell line and the limited disease annotation coverage (Fig. 4B).

We next examined the interaction networks among disease-associated genes identified by DEG and PSGRN in the K562 dataset. Both methods recovered a densely connected subnetwork enriched in ribosomal and mitochondrial genes, which are commonly observed as dominant modules in large-scale gene network analyses (Fig. 4, C and D). Shared hub genes such as RPL8, MRPS22, and PNPT1 appeared prominently in both DEG and PSGRN inferred subnetworks, reflecting the common biological signals detected by the two approaches. This overlap reinforces the consistency of PSGRN’s predictions with those from a well-established method. PSGRN incorporates not only the shared core genes but also additional regulators such as ACTB (linked to cytoskeletal remodeling and hematopoiesis), RPL5 (associated with ribosomopathies), and HSPA9 (involved in mitochondrial anemia). These additions suggest that PSGRN may provide complementary insights into the regulatory network inference, without deviating from the core architecture supported by DEG.

Overall, PSGRN consistently recovers major biological modules that overlap with those found by DEG, supporting its robustness and potential to provide complementary network information.

### PSGRN performs competitively better than other methods on noninterventional data

Although our method is primarily developed for interventional data, we further test its robustness on purely observational data. To ensure a fair and standardized comparison, we used the BEELINE pipeline, which systematically evaluates state-of-the-art (SOTA) algorithms for GRN inference from single-cell transcriptional data (*17*).

We benchmarked PSGRN and other algorithms across six single-cell RNA sequencing (scRNA-seq) datasets: Human Embryonic Stem Cells [hESC (*18*)], Human Mature Hepatocytes [hHep (*19*)], Mouse Embryonic Stem Cells [mESC (*20*)], and three subsets of Mouse Hematopoietic Stem Cells [mHSC (*21*)]—Erythroid (mHSC-E), Granulocyte-Monocyte (mHSC-GM), and Lymphoid (mHSC-L). Each dataset was processed according to the methods described in the respective publications, with log transformation applied to transcripts per kilobase million (TPM) or fragments per kilobase million (FPKM) counts where necessary. We excluded genes expressed in fewer than 10% of the cells and selected 500 and 1000 highly variable genes (HVGs) for GRN inference. For evaluation, we used three types of ground-truth networks: cell type–specific ChIP-seq, nonspecific ChIP-seq, and functional interaction networks (human and mouse STRING networks). Algorithms were compared on the basis of the area under the precision-recall curve (AUPRC).

**Fig. 5.**
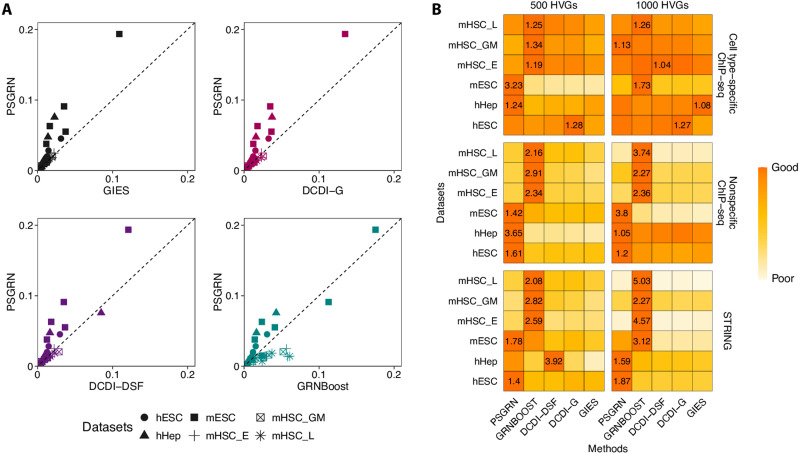
Benchmark PSGRN with the other GRN inference methods on six external datasets only containing observational data. Models were trained using two sets of HVGs (500 HVGs and 1000 HVGs) and evaluated against three gold-standard networks: cell type–specific ChIP-seq, nonspecific ChIP-seq, and the STRING database. Each method was tested across 36 evaluation scenarios (six datasets × two HVG sets × three gold standards), with performance measured by the AUPRC. (**A**) Comparison of the AUPRC values of PSGRN 5K with the other methods across all evaluation scenarios. (**B**) Summary of the relative performances of the methods in each evaluation scenario based on their AUPRC ratios (AUPRC/AUPRC of random predictor). The tile colors represent the normalized AUPRC ratios whose maximum value is 1. In each evaluation scenario, the normalized AUPRC ratios are the original value divided by the best AUPRC ratio. The numbers within the tiles display the original AUPRC ratios and are shown only when the corresponding method achieved the highest score in the given scenario.

PSGRN consistently outperformed methods such as GIES, DCDI-G, and DCDI-DSF across most datasets. When compared to GRNBoost, PSGRN delivered competitive results, with each algorithm excelling on different datasets (Fig. 5A). To better illustrate the PSGRN’s performance compared to random predictions, we also report the AUPRC ratio, which is the AUPRC divided by the performance of a random predictor. PSGRN demonstrated its robustness in capturing complex regulatory relationships in human cells by presenting superior performance on human datasets, particularly hESC and hHep. Meanwhile, GRNBoost excelled on mouse datasets, such as mESC and mHSC, suggesting that it may be better suited for GRN inference in murine models, especially in stem cell differentiation contexts (Fig. 5B).

Overall, PSGRN achieved competitive accuracy, performing on par with GRNBoost, which has been noted for its SOTA performance on observational datasets. PSGRN’s robustness across diverse datasets, including human and mouse data, underscores its versatility and reliability for GRN inference, even when working with noninterventional data.

## DISCUSSION

In this study, we developed PSGRN, a semisupervised framework for inferring GRNs using scRNA-seq data and demonstrated its robustness across a wide range of interventional and observational datasets. Our approach integrates both interventional and observational data, leveraging pseudoannotations and training to infer gene interactions. The results highlight PSGRN’s scalability, accuracy, and biological relevance, positioning it as a framework that can adapt to different data settings and improve the reliability of inferred GRNs.

One of PSGRN’s key achievements is its exceptional performance in scenarios with interventional data, a domain where many existing GRN and causal inference methods encounter difficulties. In our framework, self-training with pseudoannotations addresses these key limitations. The initial labels, which are based on expression correlations from both observational and interventional data, provide only a rough estimate of regulatory relationships. The self-training process then refines these predictions by iteratively improving the initially noisy labels. We train a predictive model using features from average gene expressions under both baseline and perturbation conditions to the model to capture higher-order patterns within the data. The threshold for defining positive gene pairs in our pseudoannotation step mainly affects performances and is the main hyperparameter we tune in the preexperiment (Fig. 1). The number 0.1 determined from on the training data aligns a commonly used correlation score (0.15) to indicate coexpression (*22*), and it is shown to be stable and generalizable to unseen perturbation arms and external datasets from different species (Fig. 5 and figs. S3 and S11). This suggests that the tuning did not overfit to the CausalBench datasets, and the performance advantages observed in our comparisons remain reliable. In addition, PSGRN is inherently capable of producing continuous probability scores for all gene pairs, which preserve more nuanced biological information than simple binary outputs.

In the benchmarking on the K562 and RPE1 cell line datasets, PSGRN demonstrated higher inference efficiency, achieving up to 80% improvement in the mean Wasserstein score and a 50% reduction in the FOR compared to other methods when more interventional data were available. The ability of PSGRN to leverage interventional data also manifests in its superior performance in recovering biologically verified interactions. Using the ground-truth datasets CORUM, STRING, and ChIP-seq data, we also demonstrated that PSGRN was capable of identifying functionally significant gene pairs with a high degree of accuracy. As interventional data were introduced, PSGRN saw substantial improvements in both precision and recall, outperforming other methods by over 60%. In addition to the original PSGRN implementation, we evaluated a variant incorporating triangle inequality–based pruning (PSGRN-prune) (*23*). The unpruned PSGRN consistently achieved higher F1 scores across all biological evaluations, whereas PSGRN-prune yielded slightly higher Wasserstein distances (fig. S10). These results suggest that pruning removes biologically supported edges but may enrich for interactions with stronger perturbation-driven effects. Therefore, we recommend using the pruned version only when the goal is to prioritize gene pairs with large perturbation effects (i.e., maximize the Wasserstein distance).

To further evaluate the model stability in edge-case settings and its potential generalizability, PSGRN was applied to the purely observational data. Under this situation, PSGRN acted more similarly to the coexpression-based method WGCNA (*24*) with its correlations refined by the gene expression levels and the trained predictive model. In the observational portions of the K562 and RPE1 datasets, PSGRN achieved competitive performances in both statistical and biological evaluations. Furthermore, on six external human and mouse single-cell datasets, PSGRN consistently performed on par with GRNBoost. The overall versatility across both human and mouse datasets, regardless of whether they contain interventional data, suggests that PSGRN is a reliable and flexible tool for GRN inference across diverse biological contexts.

However, although we observed that GRNBoost generally performed better on mouse datasets (e.g., mESC and mHSC), whereas PSGRN showed advantages on human datasets (e.g., hESC and hHep), in which the these differences could potentially reflect inherent biological variations between human and mouse GRNs between the two approaches, our current study has not systematically investigated these possibilities. This remains an open direction for future research. In addition, although PSGRN can operate using only observational data, we still suggest using PSGRN on a high-coverage perturbation dataset to fully leverage its advantages. Our rank-shift analysis further supports this point: The self-training step yields the strongest refinement of regulatory relationships when sufficient perturbation information is available, but its benefits diminish in low-intervention settings (fig. S11).

Another data-related limitation concerns the number of genes included in the dataset. Because its self-training approach relies on pseudoannotations generated from gene-gene correlations, datasets with very few genes can lead to unreliable or indistinguishable pseudolabels. For instance, in our evaluation on the BEELINE benchmark datasets—each containing only around 20 selected genes—PSGRN failed to generate informative pseudolabels as the correlation values among gene pairs were highly similar. In some cases, all pairwise correlations exceeded the predefined threshold of 0.1, resulting in insufficient separation between positive and negative examples. To further investigate the effects of sample and gene numbers, we conducted additional experiments on a yeast dataset using varying fractions of genes and samples (*25*). The performance of PSGRN declined when the number of genes or samples dropped below 1928 genes and 82 cells, respectively, highlighting its sensitivity to dataset size (fig. S9).

One future direction of further extending the PSGRN framework is to incorporate multiomics data, which has become a growing focus in single-cell biology (*26*). Beyond transcriptomic data, modalities such as chromatin accessibility (e.g., scATAC-seq) and protein expression (e.g., CITE-seq) offer complementary regulatory information that can improve the resolution and accuracy of GRN inference. For example, chromatin accessibility data can help identify active regulatory regions and potential transcription factor binding sites, whereas proteomics can reveal posttranscriptional regulation and protein-level feedback that are not captured by RNA expression alone. Recent studies, such as DIRECT-NET, CellOracle, and FigR, have demonstrated the benefits of integrating these diverse data types in uncovering more comprehensive and biologically meaningful GRNs (*27*, *28*). Incorporating such information into PSGRN could help mitigate limitations of correlation-based pseudolabeling by providing orthogonal evidence for regulatory interactions, ultimately enhancing model robustness and interpretability.

In conclusion, PSGRN advances GRN inference by combining self-training with synthetic pseudoannotation, effectively using both interventional and observational data. Its superior performance in identifying biologically meaningful interactions, along with its scalability and robustness across diverse data conditions, makes it a valuable tool for modern GRN analysis. This has practical implications in biomedical research, particularly in therapeutic discovery. For instance, PSGRN-inferred networks can be used to prioritize transcription factors driving oncogenic programs in cancer, identify master regulators in immune cell differentiation for autoimmune disease studies, or uncover potential intervention points in drug resistance pathways.

## MATERIALS AND METHODS

### Raw expression data preprocessing

The raw scRNA-seq data were organized in “h5ad” files, and the corresponding metadata were retrieved from Replogle *et al.*’s (*14*) Perturb-seq work. Raw expression data were first filtered at the perturbation level based on three criteria. For a specific perturbation experiment, we would keep the related cells if it (i) induced significant differential expression (*P* value < 0.05) of at least 50 genes determined by the Anderson-Darling test with Benjamini-Hochberg correction, (ii) had at least 25 cells remained after the QC (the cell numbers can be directly accessed from the metadata files), and (iii) achieved at least 30% knockdown on the target gene. Otherwise, all of the cells belonging to this experiment would be excluded. These “strong” perturbations (interventional data), as well as the cells for nontargeting controls (observational data), were maintained for downstream analysis.

Next, we filtered the data at the individual cell level by assessing the effectiveness of each perturbation, comparing the perturbed gene’s expression level in the interventional data with the levels in the observational data. The threshold of the expression level is at the 10th percentile of this gene in the observational data. Any cells with this gene exceeding this threshold were excluded. Note that we did not perform this filtering if the gene’s expression was not measured in the dataset.

The processed expression data were normalized by total counts over all genes for each cell and then log transformed after adding 1. The function “normalize_per_cell” in ScanPy (*29*), with its parameter “key_n_counts = ‘UMI_count’”, was used to implement this normalization.

### Retrieve the pseudoannotation

The initial gold standard indicating whether a pair of genes has a regulatory connection is determined by the correlation from their expression data (Fig. 1C, steps ① and ②).

Specifically, in the processed expression matrix *M*, for a directed gene pair $<g_{i},g_{j}>$ ($g_{i}$ affects $g_{j}$, i≠j), let i,j be the column indexes of $g_{i}$ and $g_{j}$, $I^{\prime }$ be the row indexes for all records where genes $g_{i}$ and $g_{j}$ are perturbed, and $N^{\prime }$ be the row indexes of all observational records. We first retrieved the interventional data: $M_{I^{\prime },i}$ and $M_{I^{\prime },j}$. Then, we sampled the observational data $M_{{N\_\text{sampled}}^{\prime },i}$ and $M_{{N\_\text{sampled}}^{\prime },j}$ from $M_{N^{\prime },i}$ and $M_{N^{\prime },j}$ to ensure the lengths of these observational samples matched those of the interventional data. Last, we concatenated them to form two vectors: $M_{I^{\prime }+{N\_\text{sampled}}^{\prime },i}$ and $M_{I^{\prime }+{N\_\text{sampled}}^{\prime },j}$. They are used to calculate the correlation for this gene pair. If $g_{i}$ is not available in the interventional data, the correlation will be calculated on all related observational data instead. Gene pair $<g_{i},g_{j}>$ will be assigned 1 if its absolute correlation is larger than 0.1, otherwise 0.

### Extract the features of gene pairs

Features of the gene pairs are generated from the statistics of their expression profiles (Fig. 1C, step ③). The *z*-score normalization at the row level will further normalize the processed expression matrix. Then, four features will be derived for each gene pair $<g_{i},g_{j}>$: (i) $\bar{M_{N^{\prime },i}}$, (ii) $\bar{M_{N^{\prime },j}}$, which represent the average observational expression levels of $g_{i}$ and $g_{j}$, and (iii) $\bar{M_{I^{\prime },i}}$, (iv) $\bar{M_{I^{\prime },j}}$, which represent the average expression levels of $g_{i}$ and $g_{j}$ under intervention. If the interventional data were unavailable, the last two features were filled with 0 and NaN.

### Model settings, training, and inference

We use the LightGBM (*30*) to tackle this binary classification task with the following parameters: num_leaves = 5 (max number of leaves in one tree), max_depth = 2 (max depth for tree model), min_data_in_leaf = 5 (minimal number of data in one leaf), learning_rate = 0.05, min_gain_to_split = 0.01, and num_iterations = 1000.

Under the self-training framework (*12*), the model is trained on the entire dataset, which consists of the aforementioned gene pair features and initial gold standards, and then predicts back to retrieve the inference. All of the possible gene pairs and their pseudoannotations (as initial gold standards) are used for training, and there is no separate test set. The classification model is expected to “correct” the initial gold standards with the additional information from gene expression levels and the expression changes after training. Thus, it is applied back to the same data to generate the new inferences. Only one train-predict loop is conducted in our PSGRN tool. After that, we sorted the gene pairs in descending order according to the model-inferred scores and selected the top 1000 (PSGRN 1K) or top 5000 (PSGRN 5K) gene pairs with the highest prediction scores as our final outputs.

### Evaluation metrics

We use two distinct types of metrics: a biology-driven approximation of ground truth and a statistical metric designed for model comparison following the evaluation framework of CausalBench (*10*). The biology-driven evaluation, although not entirely specific to the cell type under investigation, leverages established biological knowledge to provide additional validation. The statistical metric, in contrast, is data dependent and independent of prior knowledge, making it a robust tool for model assessment and ensuring a strong correlation with downstream applications.

#### Statistical evaluation

For an inferred gene pair <Gene A, Gene B>, where Gene A is the inferred regulator and Gene B is the target, the statistical evaluations measure the expression differences of Gene B in the observational data (observational samples) and the interventional data where Gene A is perturbed (interventional samples). The Wasserstein distance is used to quantify the differences between the two samples, and the two-tailed Mann-Whitney *U* test is used to assess its significance. If the inferred gene pair has a *P* value of < 0.05, it will be counted as true positives (TPs), otherwise false positives (FPs). With these numbers, two metrics are derived: precision, the ratio of the TPs to the total number of TP and FP, and the mean Wasserstein distance, which averages the distances among all the inferred pairs.

As relying solely on the Wasserstein distance and precision may introduce bias, where the model can overlook gene pairs with moderate regulation to achieve better performance, another metric, called FOR, is also introduced in this evaluation framework. The FOR tries to quantify the predicted absence of gene interactions. In implementation, 2000 random gene pairs are sampled from all available genes. After removing the pairs that the models infer, the remaining pairs are tested by the two-tailed Mann-Whitney *U* test, and those with a *P* value of < 0.05 are false negatives (FNs). The FOR is the number of FNs divided by 2000.

A better algorithm is expected to have a better balance between maximizing the Wasserstein distance and minimizing the FOR.

#### Biological evaluation

In the biological evaluation step, the network datasets are extracted from two widely used open biological databases: CORUM (*31*) and STRING (*32*). The CORUM database comprises experimentally validated protein complexes from mammals derived from individual experimental publications and excluding results from high-throughput experiments. The human protein complexes from the CORUM database are extracted and aggregated to form a gene network. The STRING database includes known and predicted protein-protein interactions. From STRING, we created two evaluation networks: one comprising all types of known and predicted interactions (string_network), which forms a protein-protein interaction network, and another comprising only physical interactions (string_physical), which forms a protein-protein physical interaction network.

Two other cell line–specific ChIP-seq networks are included in this evaluation. For K562, the dataset is derived from the Chip-Atlas (*33*) and ENCODE (*34*) databases. Regarding the RPE1, as it lacks extensive characterization in the existing literature, we generated networks in a comparable manner but based on the more thoroughly studied epithelial cell line, HepG2. RPE1 also belongs to the epithelial cell lineage.

To provide a more holistic evaluation, we first integrated these datasets into a Pooled Biological dataset for K562 and RPE1 by combining all the extracted data sources into a single network to collectively assess the potential gene interactions. This approach offers a comprehensive overview of interactions based on the aggregated data. Next, we developed a Pooled Biological Significant dataset as the ground-truth network, which includes only edges considered significant. This filtering is implemented by treating these gene pairs as inferences and evaluating them with statistical metrics.

In the final evaluation, the reference networks are first filtered to ensure that all the involved genes are available in our K562 and RPE1 training datasets. Then, we use precision and recall as primary performance metrics to assess the accuracy of predicted gene interactions by the algorithms. TP samples are the inferences also presented in the reference network, whereas the FP samples are the other inferences. FN samples are those pairs recorded in the reference but not detected by the algorithms. Precision can then be derived from TP/(TP + FP), and recall can be derived from TP/(TP + FN). A better algorithm is expected to maximize both the precision and recall. Therefore, we further calculate the F1 scores from precisions and recalls to determine which top-K selection provided better overall performance.

### Benchmark settings

All methods receive the same input of K562 and RPE1 with the same data preprocessing step. The parameters of the methods were set as the implementations of the CausalBench codebases. Detailed configurations of the methods are summarized below:

GRNBoost (*8*): Implemented by the “arboreto” package using the function “grnboost2” with the parameter: early_stop_window_length as 10. GRNBoost 1K set “limit” to 1000, and GRNBoost 5K set “limit” to 5000.

GIES (*35*): Implemented by the “gies” package using the function “fit_bic” with the parameters: literate as False. In addition, “expression_threshold” was set to 0.5, and “partition sizes” was set to 30.

DCDI-G (*9*): Implemented by the “dcdi” package using the class “LearnableModel_NonLinGaussANM” with the parameters: cond_n_layers = 2, and cond_hid_dim = 15. In addition, “expression_threshold” was set to 0.25, and “partition sizes” was set to 50.

DCDI-DSF (*9*): Implemented by the “dcdi” package using the class “DeepSigmoidalFlowModel” with the following parameters:cond_n_layers = 2, cond_hid_dim = 15, cond_nonlin = “leaky-relu”, flow_n_layers = 2, and flow_hid_dim = 10. In addition, “expression_threshold” was set to 0.25, and “partition sizes” was set to 50.

PC (*36*): Implemented by the “causallearn” package using the function “pc” with the default parameter setting. In addition, “expression_threshold” was set to 0.5, and “partition sizes” was set to 30.

Sortnregress (*37*): Implemented by the “scikit-learn” package using the function “LinearRegression.fit” with the default parameter setting and “LassoLarsIC.fit” setting criterion as “bic.”

DEG (*15*): Implemented by the R “DESeq2” package. Differential expression analysis was performed separately for each perturbation compared to nontargeting control cells. Perturbation-gene pairs were ranked by absolute log_2_ fold change (log_2_FoldChange) and statistical significance (*P*_adj_), with higher log_2_FoldChange and lower *P*_adj_ prioritized. The top 1000 and top 5000 perturbation-gene pairs were selected for downstream GRN prediction, corresponding to the “DEG 1K” and “DEG 5K” configurations, respectively.

MeanDifference (*16*): Self-implement following the method description. Evaluate the causal influence between a regulator X and target Y by computing the absolute difference in the mean expression of Y between observational data and data from interventions targeting X. This metric directly captures the expression change in Y attributable to perturbing X. Gene pairs are ranked by the magnitude of this difference, and the top 1K and 5K pairs were selected as predictions.

MixScale (*38*): Implemented by the R “Mixscale” package. For GRN inference, perturbation-specific differential expression analysis was performed using the Mixscale score weighted model (Run_wmvRegDE). Genes were ranked by the absolute log fold change (logFC) and statistical significance (adjusted *P* value < 0.05), with higher logFC and lower *P* value prioritized. The top 1000 and top 5000 gene-perturbation pairs were selected for GRN prediction, corresponding to the “MixScale 1K” and “MixScale 5K” configurations, respectively.

### BEELINE experimental scRNA-seq datasets

We obtained four additional scRNA-seq datasets from the BEELINE pipeline for a more comprehensive benchmark, comprising two datasets from mice and two from humans (*17*). There were a total of six cell types across these datasets. We preprocessed each dataset using the procedure described in the corresponding paper. We additionally filtered out any genes expressed in fewer than 10% of the cells. Here are the key details of the datasets after removing the pseudotime computation details:

Mouse Hematopoietic Stem Cells (mHSCs) (*21*): The dataset includes normalized expression data for 1656 hematopoietic stem and progenitor cells (HSPCs) across 4773 genes, sourced from supplementary data provided by the authors. GRNs were inferred for three lineages: erythroid, granulocyte-monocyte, and lymphoid.

Mouse embryonic stem cells (mESC) (*20*): This dataset contains scRNA-seq expression measurements for 421 primitive endoderm (PrE) cells differentiated from mESCs, collected at five distinct time points (0, 12, 24, 48, and 72 hours).

Human Mature Hepatocytes (hHEP) (*19*): This dataset is derived from a scRNA-seq experiment on induced pluripotent stem cells (iPSCs) in two-dimensional culture, which differentiates into hepatocyte-like cells. It includes 425 scRNA-seq measurements taken at multiple time points: days 0 (iPSCs), 6, 8, 14, and 21 (mature hepatocyte-like cells).

Human Embryonic Stem Cells (hESC) (*18*): This dataset comprises 758 cells measured in a time-course scRNA-seq experiment, which followed the differentiation of human embryonic stem cells into definitive endoderm cells at six time points: 0, 12, 24, 36, 72, and 96 hours.

### BEELINE ground-truth network collection and processing

For our analysis, we used three types of ground-truth datasets provided by BEELINE. This approach allowed us to evaluate the performance of GRN methods across different levels of regulatory evidence and interaction types.

Cell type–specific dataset: For each experimental scRNA-seq dataset, it gathered ChIP-seq data from the ENCODE, ChIP-Atlas, and ESCAPE databases, prioritizing data from the same or closely related cell types.

Nonspecific dataset: It used DoRothEA (*39*), RegNetwork (*40*), and TRRUST (*41*), a comprehensive database that integrates ChIP-seq and transcriptional regulatory information from various sources.

Functional dataset: It incorporated the STRING (*32*) networks for humans and mice, which capture functional interactions that may not necessarily be transcriptional.

### BEELINE evaluation

We used a standardized evaluation pipeline from BEELINE across all datasets in this study to ensure consistent comparison between algorithms. First, we selected genes for GRN inference by using the “scanpy.pp.highly_variable_genes” function from the “scanpy” package. This allowed us to identify 500 and 1000 HVGs, which were then used as input data for our model. These selected genes and their gene expression data were fed into the PSGRN model.

Because the datasets provided by the BEELINE pipeline do not contain intervention data, we set the intervention fraction to 0 in our PSGRN implementation. The PSGRN model then inferred a prediction network consisting of 5000 ranked gene pairs.

Next, we evaluated the predicted network using the BEELINE pipeline. Specifically, we used BEELINE’s evaluation framework to compute the AUPRC. In this evaluation, the true edges in the relevant ground-truth networks were treated as the “ground truth,” whereas the ranked edges produced by our algorithm were considered as predictions. The evaluation was performed across three datasets: Cell type–specific ChIP-seq data, Nonspecific ChIP-seq data, and STRING. This comprehensive evaluation provided a robust measure of our model’s performance, with AUPRC scores summarizing the accuracy of predictions in terms of both precision and recall.

We applied the same evaluation process to all other benchmark algorithms under consideration to ensure a fair comparison. Each algorithm’s prediction network was similarly evaluated using BEELINE’s framework across the same datasets, allowing us to consistently assess and compare performance across various methods.

## References

[1] K. Akers, T. M. Murali, Gene regulatory network inference in single-cell biology. Curr. Opin. Syst. Biol. 26, 87–97 (2021).

[2] X. Hu, Y. Hu, F. Wu, R. W. T. Leung, J. Qin, Integration of single-cell multi-omics for gene regulatory network inference. Comput. Struct. Biotechnol. J. 18, 1925–1938 (2020). 32774787 10.1016/j.csbj.2020.06.033 PMC7385034

[3] A. Dixit, O. Parnas, B. Li, J. Chen, C. P. Fulco, L. Jerby-Arnon, N. D. Marjanovic, D. Dionne, T. Burks, R. Raychowdhury, B. Adamson, T. M. Norman, E. S. Lander, J. S. Weissman, N. Friedman, A. Regev, Perturb-Seq: Dissecting molecular circuits with scalable single-cell RNA profiling of pooled genetic screens. Cell 167, 1853–1866.e17 (2016). 27984732 10.1016/j.cell.2016.11.038 PMC5181115

[4] P. Datlinger, A. F. Rendeiro, C. Schmidl, T. Krausgruber, P. Traxler, J. Klughammer, L. C. Schuster, A. Kuchler, D. Alpar, C. Bock, Pooled CRISPR screening with single-cell transcriptome readout. Nat. Methods 14, 297–301 (2017). 28099430 10.1038/nmeth.4177 PMC5334791

[5] P. Datlinger, A. F. Rendeiro, T. Boenke, M. Senekowitsch, T. Krausgruber, D. Barreca, C. Bock, Ultra-high-throughput single-cell RNA sequencing and perturbation screening with combinatorial fluidic indexing. Nat. Methods 18, 635–642 (2021). 34059827 10.1038/s41592-021-01153-z PMC7612019

[6] V. A. Huynh-Thu, A. Irrthum, L. Wehenkel, P. Geurts, Inferring regulatory networks from expression data using tree-based methods. PLOS ONE 5, e12776 (2010). 20927193 10.1371/journal.pone.0012776 PMC2946910

[7] S. Aibar, C. B. González-Blas, T. Moerman, J. Wouters, V. A. Huynh-Thu, H. Imrichova, Z. K. Atak, G. Hulselmans, M. Dewaele, F. Rambow, P. Geurts, J. Aerts, J.-C. Marine, J. van den Oord, S. Aerts, SCENIC: Single-cell regulatory network inference and clustering. Nat. Methods 14, 1083–1086 (2017). 28991892 10.1038/nmeth.4463 PMC5937676

[8] T. Moerman, S. A. Santos, C. B. González-Blas, J. Simm, Y. Moreau, J. Aerts, S. Aerts, GRNBoost2 and Arboreto: Efficient and scalable inference of gene regulatory networks. Bioinformatics 35, 2159–2161 (2019). 30445495 10.1093/bioinformatics/bty916

[9] P. Brouillard, S. Lachapelle, A. Lacoste, S. Lacoste-Julien, A. Drouin, Differentiable causal discovery from interventional data. arXiv:2007.01754 [cs.LG] (2020).

[10] M. Chevalley, Y. Roohani, A. Mehrjou, J. Leskovec, P. Schwab, CausalBench: A large-scale benchmark for network inference from single-cell perturbation data. arXiv:2210.17283 [cs.LG] (2022). 10.1038/s42003-025-07764-y PMC11897147 40069299

[11] A. R. Chapman, D. F. Lee, W. Cai, W. Ma, X. Li, W. Sun, X. S. Xie, Correlated gene modules uncovered by high-precision single-cell transcriptomics. Proc. Natl. Acad. Sci. U.S.A. 119, e2206938119 (2022). 36508663 10.1073/pnas.2206938119 PMC9907105

[12] M.-R. Amini, F. Vasilii, L. Pauletto, L. Hadjadj, E. Devijver, Y. Maximov, Self-training: A survey. arXiv:2202.12040 [cs.LG] (2024).

[13] M. Chevalley, J. Sackett-Sanders, Y. Roohani, P. Notin, A. Bakulin, D. Brzezinski, K. Deng, Y. Guan, J. Hong, M. Ibrahim, W. Kotlowski, M. Kowiel, P. Misiakos, A. Nazaret, M. Püschel, C. Wendler, A. Mehrjou, P. Schwab, The CausalBench challenge: A machine learning contest for gene network inference from single-cell perturbation data. arXiv:2308.15395 [cs.LG] (2023).

[14] J. M. Replogle, R. A. Saunders, A. N. Pogson, J. A. Hussmann, A. Lenail, A. Guna, L. Mascibroda, E. J. Wagner, K. Adelman, G. Lithwick-Yanai, N. Iremadze, F. Oberstrass, D. Lipson, J. L. Bonnar, M. Jost, T. M. Norman, J. S. Weissman, Mapping information-rich genotype-phenotype landscapes with genome-scale Perturb-seq. Cell 185, 2559–2575.e28 (2022). 35688146 10.1016/j.cell.2022.05.013 PMC9380471

[15] M. I. Love, W. Huber, S. Anders, Moderated estimation of fold change and dispersion for RNA-seq data with DESeq2. Genome Biol. 15, 550 (2014). 25516281 10.1186/s13059-014-0550-8 PMC4302049

[16] M. Kowiel, W. Kotlowski, D. Brzezinski, “CausalBench Challenge: Differences in mean expression” (2023); https://openreview.net/forum?id=Cx9B85IlEVR.

[17] A. Pratapa, A. P. Jalihal, J. N. Law, A. Bharadwaj, T. M. Murali, Benchmarking algorithms for gene regulatory network inference from single-cell transcriptomic data. Nat. Methods 17, 147–154 (2020). 31907445 10.1038/s41592-019-0690-6 PMC7098173

[18] L.-F. Chu, N. Leng, J. Zhang, Z. Hou, D. Mamott, D. T. Vereide, J. Choi, C. Kendziorski, R. Stewart, J. A. Thomson, Single-cell RNA-seq reveals novel regulators of human embryonic stem cell differentiation to definitive endoderm. Genome Biol. 17, 173 (2016). 27534536 10.1186/s13059-016-1033-x PMC4989499

[19] J. G. Camp, K. Sekine, T. Gerber, H. Loeffler-Wirth, H. Binder, M. Gac, S. Kanton, J. Kageyama, G. Damm, D. Seehofer, L. Belicova, M. Bickle, R. Barsacchi, R. Okuda, E. Yoshizawa, M. Kimura, H. Ayabe, H. Taniguchi, T. Takebe, B. Treutlein, Multilineage communication regulates human liver bud development from pluripotency. Nature 546, 533–538 (2017). 28614297 10.1038/nature22796

[20] T. Hayashi, H. Ozaki, Y. Sasagawa, M. Umeda, H. Danno, I. Nikaido, Single-cell full-length total RNA sequencing uncovers dynamics of recursive splicing and enhancer RNAs. Nat. Commun. 9, 619 (2018). 29434199 10.1038/s41467-018-02866-0 PMC5809388

[21] S. Nestorowa, F. K. Hamey, B. Pijuan Sala, E. Diamanti, M. Shepherd, E. Laurenti, N. K. Wilson, D. G. Kent, B. Göttgens, A single-cell resolution map of mouse hematopoietic stem and progenitor cell differentiation. Blood 128, e20–e31 (2016). 27365425 10.1182/blood-2016-05-716480 PMC5305050

[22] Metware Biotechnology Inc., Understanding WGCNA Analysis in Publications, https://www.metwarebio.com/understanding-wgcna-analysis-in-publications/.

[23] D. L. Poole, A. K. Mackworth, *Artificial Intelligence* (Cambridge Univ. Press, 2023).

[24] P. Langfelder, S. Horvath, WGCNA: An R package for weighted correlation network analysis. BMC Bioinformatics 9, 559 (2008). 19114008 10.1186/1471-2105-9-559 PMC2631488

[25] M. Stock, N. Popp, J. Fiorentino, A. Scialdone, Topological benchmarking of algorithms to infer gene regulatory networks from single-cell RNA-seq data. Bioinformatics 40, btae267 (2024). 38627250 10.1093/bioinformatics/btae267 PMC11096270

[26] A. Baysoy, Z. Bai, R. Satija, R. Fan, The technological landscape and applications of single-cell multi-omics. Nat. Rev. Mol. Cell Biol. 24, 695–713 (2023). 37280296 10.1038/s41580-023-00615-w PMC10242609

[27] K. Kamimoto, B. Stringa, C. M. Hoffmann, K. Jindal, L. Solnica-Krezel, S. A. Morris, Dissecting cell identity via network inference and in silico gene perturbation. Nature 614, 742–751 (2023). 36755098 10.1038/s41586-022-05688-9 PMC9946838

[28] V. K. Kartha, F. M. Duarte, Y. Hu, S. Ma, J. G. Chew, C. A. Lareau, A. Earl, Z. D. Burkett, A. S. Kohlway, R. Lebofsky, J. D. Buenrostro, Functional inference of gene regulation using single-cell multi-omics. Cell Genom. 2, 100166 (2022). 36204155 10.1016/j.xgen.2022.100166 PMC9534481

[29] F. A. Wolf, P. Angerer, F. J. Theis, SCANPY: Large-scale single-cell gene expression data analysis. Genome Biol. 19, 15 (2018). 29409532 10.1186/s13059-017-1382-0 PMC5802054

[30] G. Ke, Q. Meng, T. Finley, T. Wang, W. Chen, W. Ma, Q. Ye, T.-Y. Liu, “LightGBM: A highly efficient gradient boosting decision tree,” in *Advances in Neural Information Processing Systems 30*, I. Guyon, U. V. Luxburg, S. Bengio, H. Wallach, R. Fergus, S. Vishwanathan, R. Garnett, Eds. (Curran Associates Inc., 2017), pp. 3146–3154.

[31] G. Tsitsiridis, R. Steinkamp, M. Giurgiu, B. Brauner, G. Fobo, G. Frishman, C. Montrone, A. Ruepp, CORUM: The comprehensive resource of mammalian protein complexes-2022. Nucleic Acids Res. 51, D539–D545 (2023). 36382402 10.1093/nar/gkac1015 PMC9825459

[32] D. Szklarczyk, R. Kirsch, M. Koutrouli, K. Nastou, F. Mehryary, R. Hachilif, A. L. Gable, T. Fang, N. T. Doncheva, S. Pyysalo, P. Bork, L. J. Jensen, C. von Mering, The STRING database in 2023: Protein-protein association networks and functional enrichment analyses for any sequenced genome of interest. Nucleic Acids Res. 51, D638–D646 (2023). 36370105 10.1093/nar/gkac1000 PMC9825434

[33] Z. Zou, T. Ohta, F. Miura, S. Oki, ChIP-Atlas 2021 update: A data-mining suite for exploring epigenomic landscapes by fully integrating ChIP-seq, ATAC-seq and Bisulfite-seq data. Nucleic Acids Res. 50, W175–W182 (2022). 35325188 10.1093/nar/gkac199 PMC9252733

[34] C. A. Davis, B. C. Hitz, C. A. Sloan, E. T. Chan, J. M. Davidson, I. Gabdank, J. A. Hilton, K. Jain, U. K. Baymuradov, A. K. Narayanan, K. C. Onate, K. Graham, S. R. Miyasato, T. R. Dreszer, J. S. Strattan, O. Jolanki, F. Y. Tanaka, J. M. Cherry, The Encyclopedia of DNA elements (ENCODE): Data portal update. Nucleic Acids Res. 46, D794–D801 (2018). 29126249 10.1093/nar/gkx1081 PMC5753278

[35] A. Hauser, P. Bühlmann, Characterization and greedy learning of interventional Markov equivalence classes of directed acyclic graphs. arXiv:1104.2808 [stat.ME] (2011).

[36] Y. Zheng, B. Huang, W. Chen, J. Ramsey, M. Gong, R. Cai, S. Shimizu, P. Spirtes, K. Zhang, Causal-learn: Causal discovery in Python. arXiv:2307.16405 [cs.LG] (2023).

[37] A. G. Reisach, C. Seiler, S. Weichwald, “Beware of the simulated DAG! causal discovery benchmarks may be easy to game,” in *NIPS ’21: Proceedings of the 35th International Conference on Neural Information Processing Systems* (Curran Associates Inc., 2024), pp. 27772–27784.

[38] L. Jiang, C. Dalgarno, E. Papalexi, I. Mascio, H.-H. Wessels, H. Yun, N. Iremadze, G. Lithwick-Yanai, D. Lipson, R. Satija, Systematic reconstruction of molecular pathway signatures using scalable single-cell perturbation screens. Nat. Cell Biol. 27, 505–517 (2025). 40011560 10.1038/s41556-025-01622-z PMC12083445

[39] L. Garcia-Alonso, C. H. Holland, M. M. Ibrahim, D. Turei, J. Saez-Rodriguez, Benchmark and integration of resources for the estimation of human transcription factor activities. Genome Res. 29, 1363–1375 (2019). 31340985 10.1101/gr.240663.118 PMC6673718

[40] Z.-P. Liu, C. Wu, H. Miao, H. Wu, RegNetwork: An integrated database of transcriptional and post-transcriptional regulatory networks in human and mouse. Database 2015, bav095 (2015). 26424082 10.1093/database/bav095 PMC4589691

[41] H. Han, J.-W. Cho, S. Lee, A. Yun, H. Kim, D. Bae, S. Yang, C. Y. Kim, M. Lee, E. Kim, S. Lee, B. Kang, D. Jeong, Y. Kim, H.-N. Jeon, H. Jung, S. Nam, M. Chung, J.-H. Kim, I. Lee, TRRUST v2: An expanded reference database of human and mouse transcriptional regulatory interactions. Nucleic Acids Res. 46, D380–D386 (2018). 29087512 10.1093/nar/gkx1013 PMC5753191

