# Supplementary material for: PSGRN: Gene regulatory network inference from single-cell perturbational data through self-training with synthetic gold standards
Source: Sci Adv. 2026 Apr 29;12(18):eaeb3376. doi: 10.1126/sciadv.aeb3376 (PMC13127566; doi:10.1126/sciadv.aeb3376)
Supplement: Supplementary file 1 — Figs. S1 to S11 Tables S1 to S4 [file sciadv.aeb3376_sm.pdf]

Science Advances
AAAS

Supplementary Materials for

PSGRN: Gene regulatory network inference from single-cell perturbational data through self-training with synthetic gold standards

Xinhan Song *et al.*

Corresponding author: Yuanfang Guan, gyuanfan@umich.edu

Sci. Adv. **12**, eaeb3376 (2026)
DOI: 10.1126/sciadv.aeb3376

This PDF file includes:

Figs. S1 to S11
Tables S1 to S4

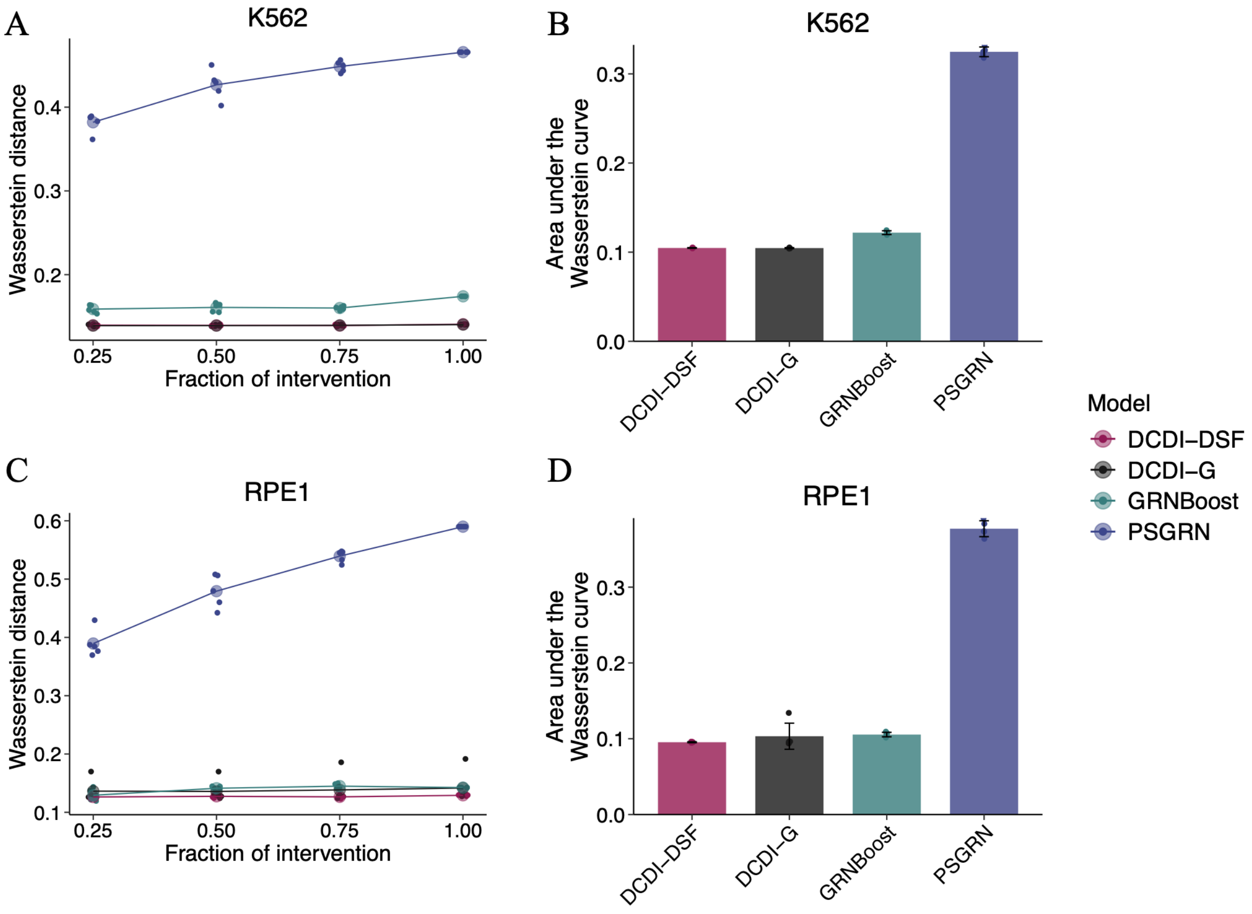

Figure S1. The Wasserstein curves and the area under the Wasserstein curves of PSGRN and the other methods. This figure compares PSGRN with the optimal hyperparameters we presented in Figure 1 and the other CausalBench baseline methods, including GRNBoost and the DCDIs. Both PSGRN and GRNBoost select the top 1000 inferred gene pairs. Similar to Figures 1D and 1E, (A, C) show the Wasserstein distances under different fractions of available interventional data. The line and the larger dots represent the average values of five repeated experiments with different random seeds. The small dots are the values of these experiments. DCDI-DSF and DCDI-G perform similarly. Their curves and dots nearly overlap. (B, D) show the areas under these Wasserstein distance curves. The heights of the bars are the average values, and the error bars are the standard deviations of these values.

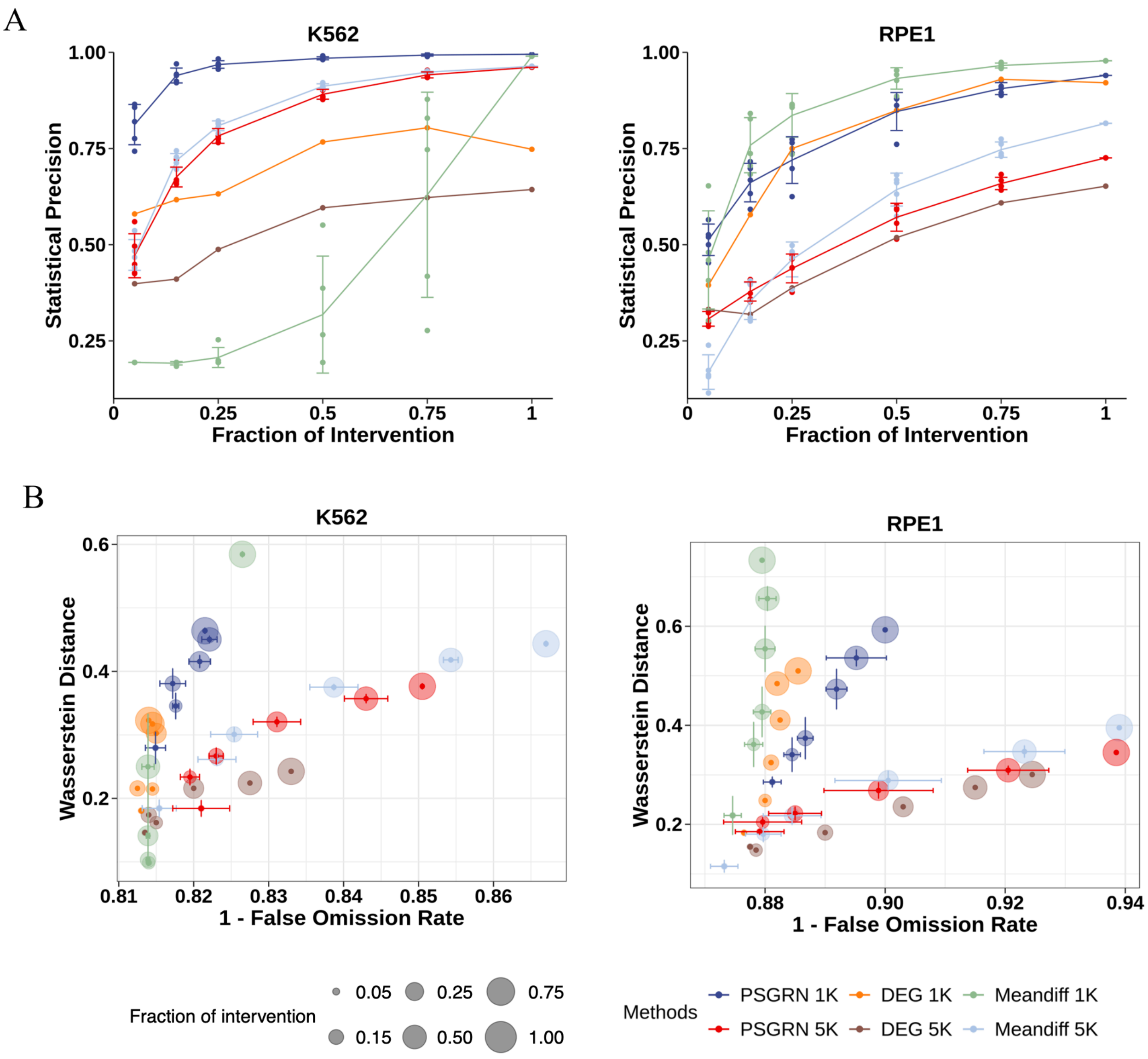

Figure S2. Benchmarking PSGRN against DEG and MeanDifference across different fractions of the interventional data and the entire datasets. (A) shows how statistical precision of PSGRN (1K and 5K) and six other algorithms changes with increasing fractions of interventional data for the K562 (left) and RPE1 (right) datasets. Each point represents the mean precision across six random seeds. (B) visualizes the mean Wasserstein distance and 1 - FOR of PSGRN (top 1K and top 5K) and six other algorithms across increasing fractions of interventional data for the K562 (left) and RPE1 (right) datasets.

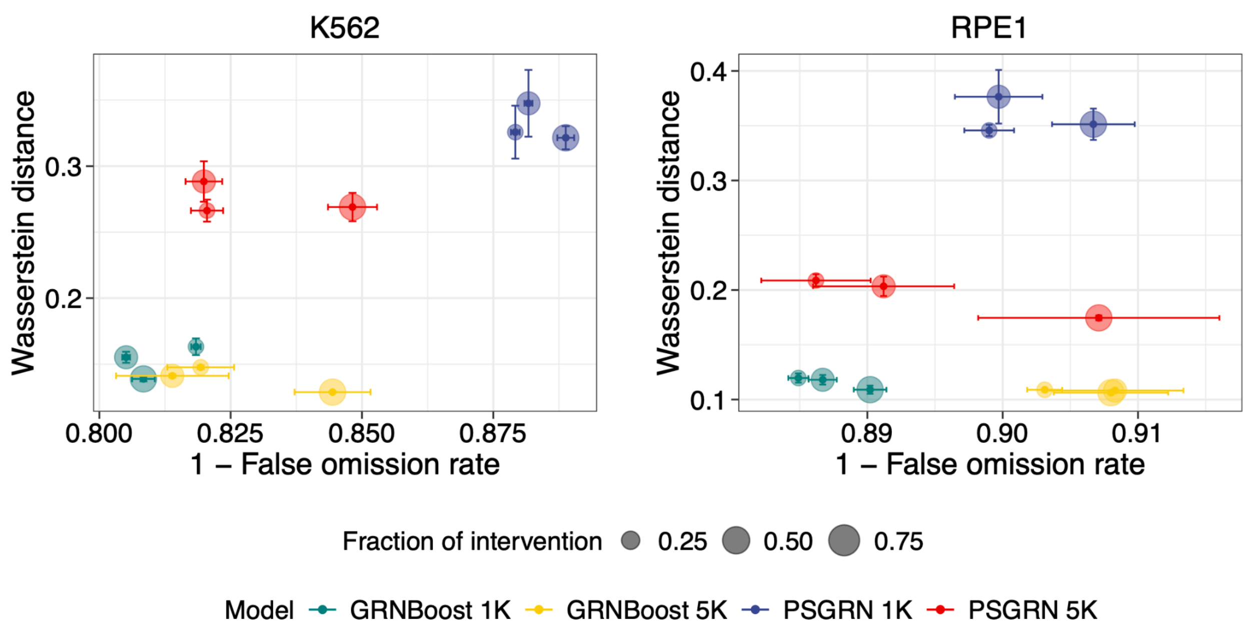

Figure S3. The statistical evaluations on the unseen interventional arms. PSGRN 1K, 5K, and GRNBoost 1K, 5K are compared under this framework. They are trained on 25%, 50% and 75% of the interventional data and evaluated on the remaining perturbations which the models have not seen. Each model is evaluated with five repetitions. The position of the point represents the average values, while the error bars represent the standard deviations.

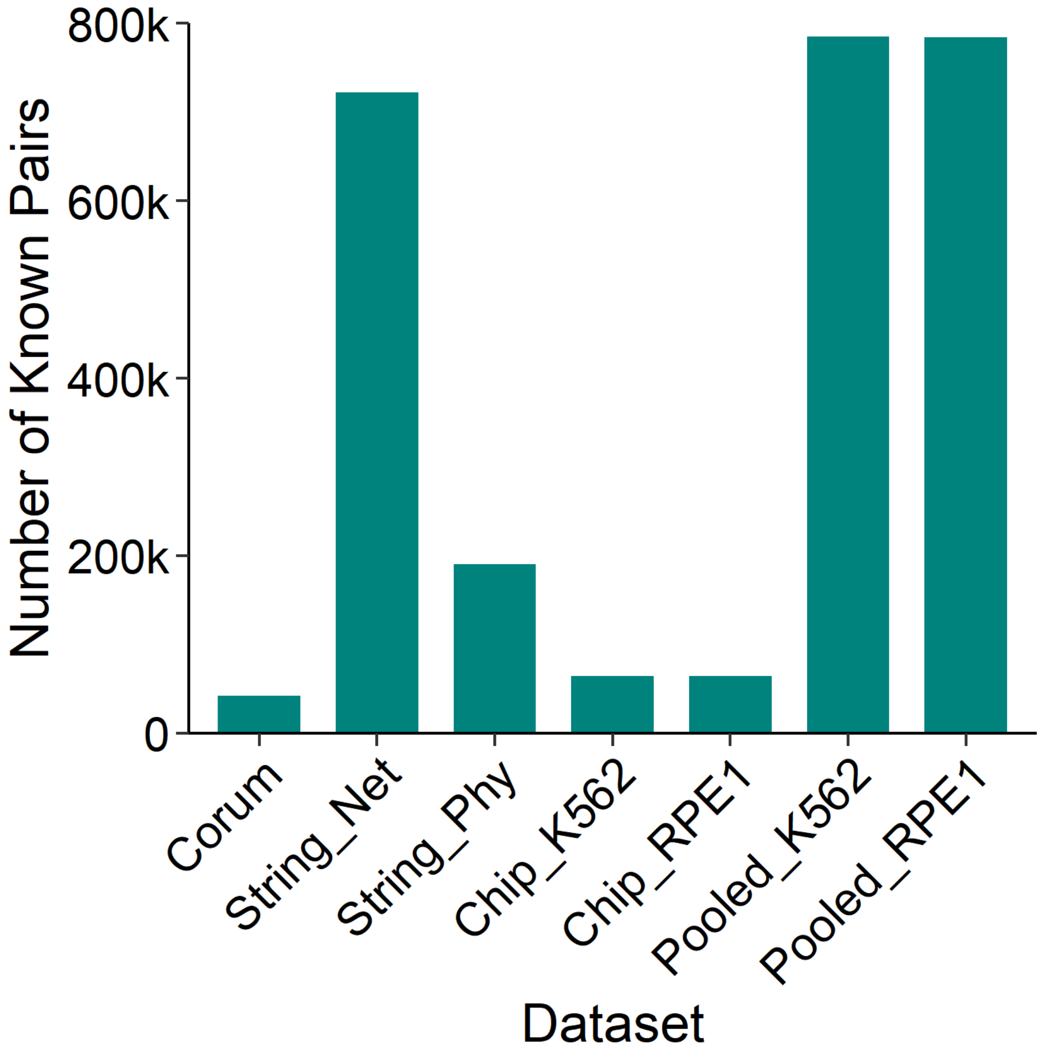

Figure S4. The number of known gene pairs in five different databases and two pooled databases. Abbreviations: String_Net, String_Network; String_Phy, String_Physical; Chip_K562, CHIP-Seq_K562; Chip_RPE1, CHIP-Seq_RPE1; Pooled_K562, Pooled Pairs of K562; Pooled_RPE1, Pooled Pairs of RPE1.

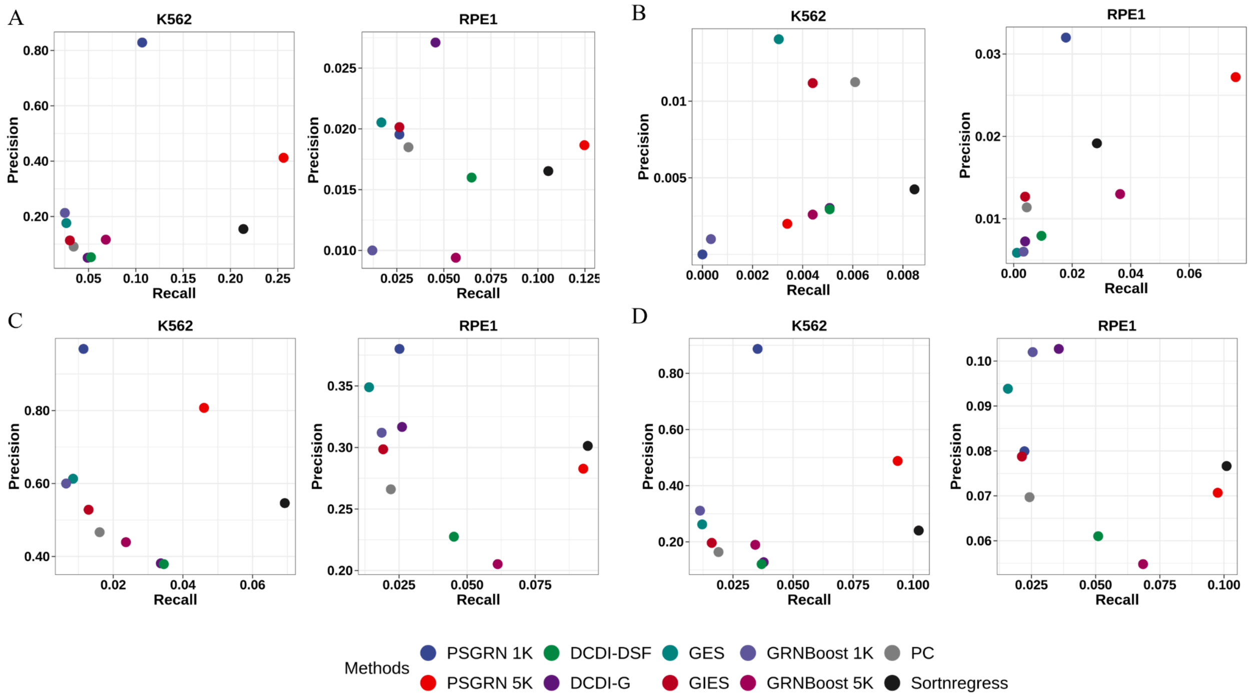

Figure S5. Benchmark PSGRN with the other GRN inference methods using biological evaluation metrics on observational data across different datasets. Figures show the biological precision and recall performance of PSGRN (top 1k and top 5k) compared to seven other algorithms on K562 and RPE1 using different evaluation datasets. (A) CORUM (B) CHIP-Seq (C) String_network (D) String_physical

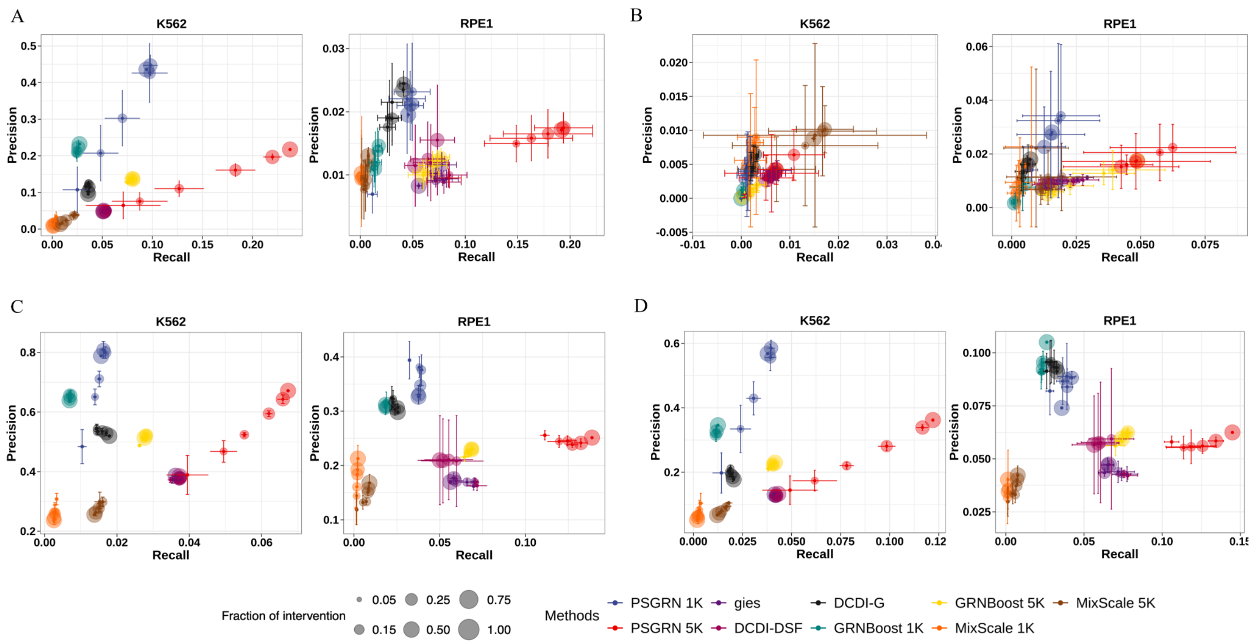

Figure S6. Benchmark PSGRN with the other GRN inference methods using biological evaluation metrics on increasing fractions of interventional data across different datasets. Figures illustrate the effect of increasing fractions of interventional data on the biological precision and recall of PSGRN (top 1k and top 5k) and five other algorithms for the K562 and RPE1 datasets using four different evaluation datasets. Each point represents the mean precision from four independent experiments conducted for each intervention fraction. (A) CORUM (B) CHIP-Seq (C) String_network (D) String_physical.

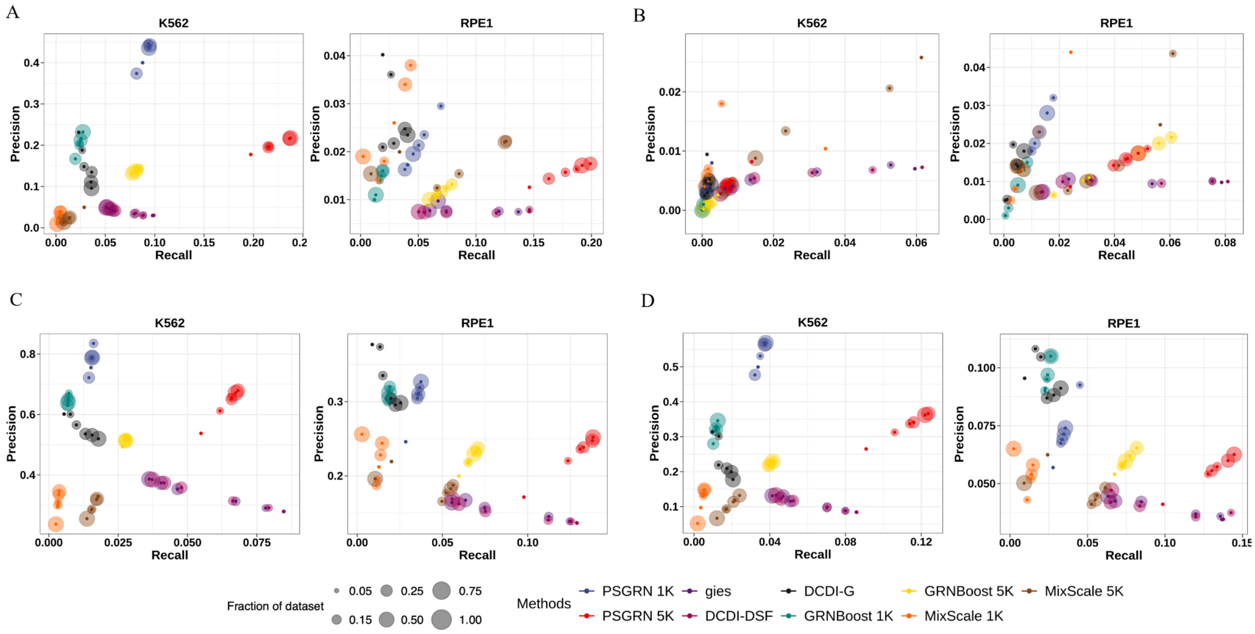

Figure S7: Benchmark PSGRN with the other GRN inference methods using biological evaluation metrics to increase dataset size fractions across different datasets. The plots demonstrate the impact of increasing dataset sizes on the biological precision and recall of PSGRN (top 1k and top 5k) and five other algorithms for the K562 (left) and RPE1 (right) datasets using four different evaluation datasets. Each point corresponds to the mean precision from four independent experiments for each dataset size. (A) CORUM (B) CHIP-Seq (C) String_network (D) String_physical.

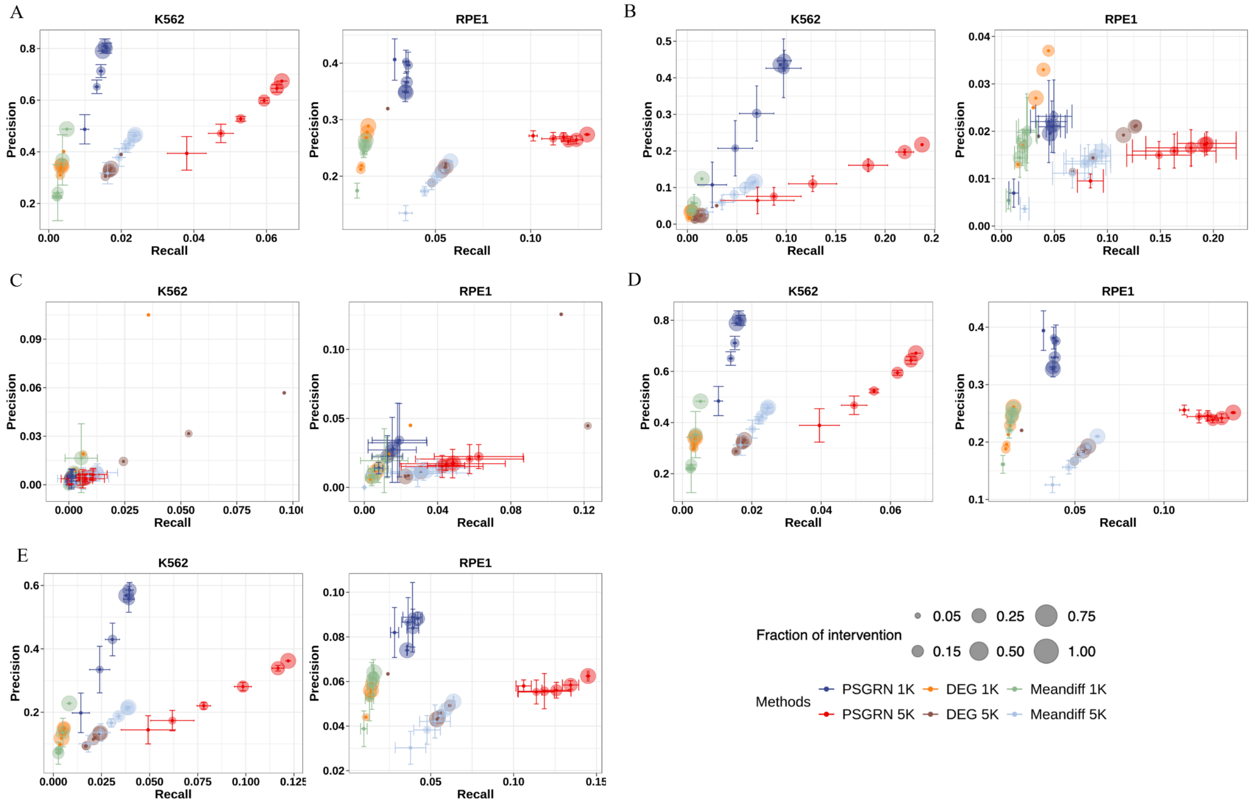

Figure S8. Benchmark PSGRN with DEG and MeanDifference on the biological evaluation metrics trained with different fractions of intervention and dataset. The figures illustrate the effect of increasing fractions of interventional data on the biological precision and recall of PSGRN (top 1K and top 5K) compared with DEG, MeanDifference for the K562 and RPE1 datasets, evaluated on five different benchmark datasets. Each point represents the mean precision from five independent experiments for each intervention fraction.(A) Pooled Pair dataset, which is created by integrating the four datasets from B, C, D and E. (B) CORUM (C) CHIP-Seq (D) String_network (E) String_physical

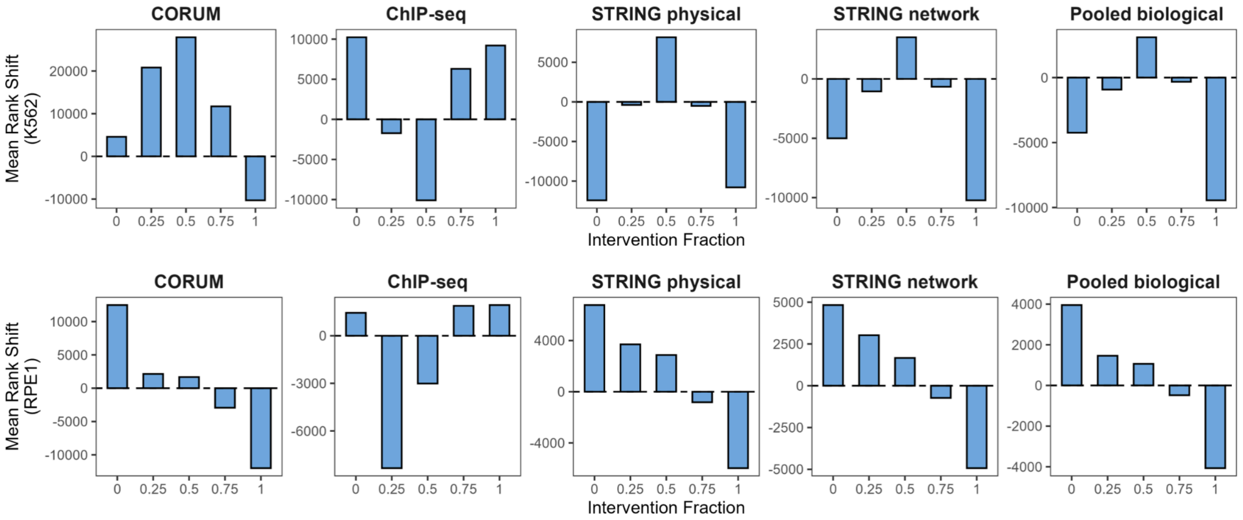

Figure S9. Mean rank shifts of ground-truth gene pairs before and after self-training. Rank shifts were computed by subtracting the initial correlation-based rank from the PSGRN-predicted rank; negative values indicate improvement in rank. Each column shows the mean shift across all ground-truth pairs for different intervention fractions and each row represents one cell line. Larger negative values reflect stronger refinement by the self-training model.

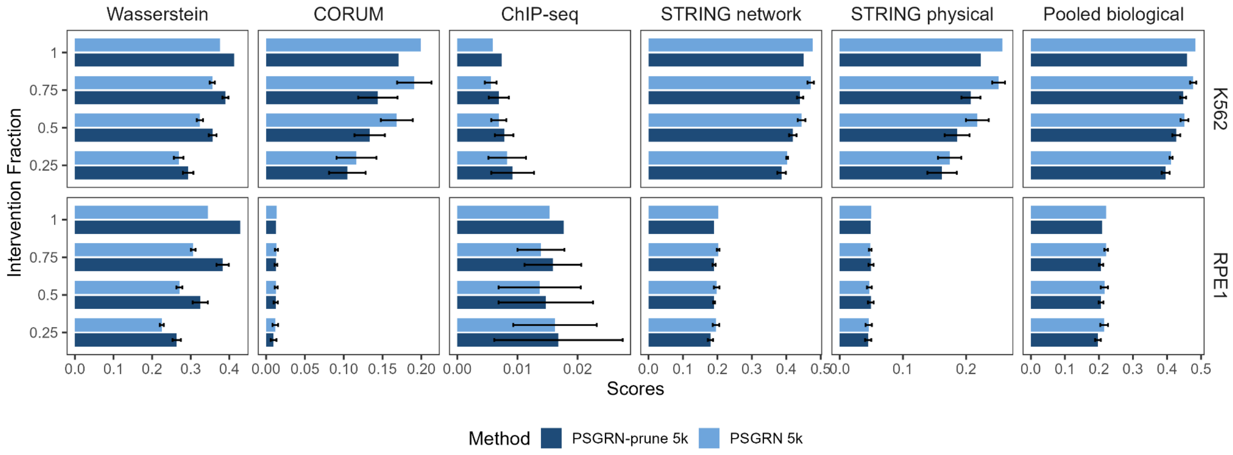

Figure S10. Compare the performances between original PSGRN and PSGRN with pruning. For the biological evaluations (CORUM, ChIP-seq, STRINGs, and Pooled biological), the scores are AUPRCs, and for “Wasserstein”, the scores are wasserstein distances. For the intervention fractions less than 1, the bars are the averaged scores of 5 repeated experiments of different seed and the error bars are the standard deviations of these scores.

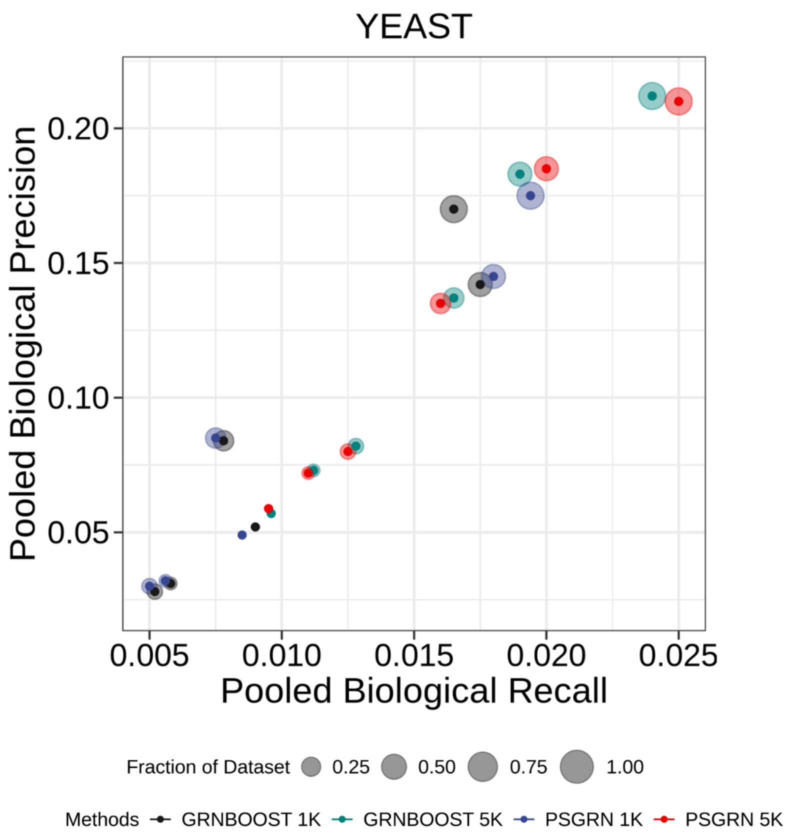

Figure S11. Biological evaluation of PSGRN and GRNBoost on the yeast dataset under varying data scales. The figure shows the impact of increasing dataset sizes (measured as fractions of the full dataset) on the pooled biological precision and recall of PSGRN (top 1k and top 5k) and GRNBOOST (top 1k and top 5k). Each point corresponds to the mean performance across four independent experiments under a specific dataset fraction. Larger point sizes indicate larger fractions of the dataset used.

Table S1. Performance in AUCs of different PSGRN hyperparameter settings with 1 randomly sampled data

K or T*	Factors of negative samples**	Norm method	K562	RPE1
Top 1000 absolute correlation (baseline)			0.2890	0.3397
500	2	/	0.1861	0.3040
2000	2	/	0.2393	0.3352
5000	2	/	0.2524	0.3552
7000	3	/	0.2684	0.3608
7000	AllNeg	/	0.2826	0.3846
7000	AllNeg	Z-score	0.3023	0.3744
7000	AllNeg	Quantile	0.2843	0.3768
0.1	AllNeg	Z-score	0.3148	/
0.2	AllNeg	Z-score	0.3027	/

* First K (the integer numbers) top-correlated gene pairs or those with correlation > T (the float numbers) are the initial positive samples

** If it is a number, it means to select (this number * # of positive samples) negative samples to train. “AllNeg” means to select all the negative samples.

Table S2. Performance in AUCs of different PSGRN hyperparameter settings with 5 randomly sampled data

K or T	Factors of negative samples	Norm method	K562	RPE1
Top 1000 absolute correlation (baseline)			0.2926 ± 0.001	0.3255 ± 0.008
5000	AllNeg	/	0.2934 ± 0.007	0.3696 ± 0.003
5000	AllNeg	Z-score	0.3053 ± 0.006	0.3642 ± 0.008
5000	AllNeg	Quantile	0.2992 ± 0.009	0.3644 ± 0.009
7000	AllNeg	/	0.2937 ± 0.003	0.3683 ± 0.01
0.1	AllNeg	Z-score	0.3248 ± 0.005	0.3771 ± 0.01
0.2	AllNeg	Z-score	0.3127 ± 0.002	0.3591 ± 0.008

Table S3. Filtering Summary for K562

Criterion	Cells Removed	Genes Removed	Perturbations Removed	Remaining Cells	Remaining Genes	Remaining Perturbations*
Raw data	–	–	–	310,385	8,563	2,058
Strong perturbation filtering	117,737	0	965	192,648	8,563	1,093
Remove cells with perturbed gene expression > 10th percentile	29,897	0	0	162,751	8,563	1,093
Keep perturbations with ≥100 cells;<br>Label the unsatisfied perturbations as “excluded”	0	7,941	471	162,751	622	622
Remove “excluded” perturbation labels;<br>Select only the effectively perturbed genes	30,179	0	0	132,572	622	622

* Include one “non-targeting” for observational data

Table S4. Filtering Summary for RPE1

Criterion	Cells Removed	Genes Removed	Perturbations Removed	Remaining Cells	Remaining Genes	Remaining Perturbations*
Raw data	–	–	–	247,914	8,749	2,394
Strong perturbation filtering	72,516	0	850	175,398	8,749	1,544
Remove cells with perturbed gene expression > 10th percentile	12,665	0	0	162,733	8,749	1,544
Keep perturbations with ≥100 cells;<br>Label the unsatisfied perturbations as “excluded”	0	0	1,161	162,733	8,749	383

Remove “excluded” perturbation labels; Select only the effectively perturbed genes	61,480	8,366	0	101,253	383	383

* Include one “non-targeting” for observational data
